# Development of a determination method for quality control markers utilizing metabolic profiling and its application on processed *Zingiber officinale* Roscoe rhizome

**DOI:** 10.1007/s11418-024-01837-8

**Published:** 2024-08-03

**Authors:** Tomohisa Kanai, Tatsuya Shirahata, Shunsuke Nakamori, Yota Koizumi, Eiichi Kodaira, Noriko Sato, Hiroyuki Fuchino, Noriaki Kawano, Nobuo Kawahara, Takayuki Hoshino, Kayo Yoshimatsu, Yoshinori Kobayashi

**Affiliations:** 1 School of Pharmacy, Kitasato University, 5–9–1 Shirokane, Minato-Ku, Tokyo, 108–8641 Japan; 2 Oriental Medicine Therapy Center, Kitasato Institute Hospital, Kitasato University, 5–9–1 Shirokane, Minato-Ku, Tokyo, 108–8641 Japan; 3 Research Center for Medicinal Plant Resources, National Institutes of Biomedical Innovation, Health and Nutrition, 1–2 Hachimandai, Tsukuba, Ibaraki 305–0843 Japan; 4 The Kochi Prefectural Makino Botanical Garden, Godaisan, Kochi, 781–8125 Japan

**Keywords:** *Zingiber officinale* Roscoe, Orthogonal Partial Least Squares (OPLS), Processing, ^1^H-NMR, GC-MS

## Abstract

This study established an Orthogonal Partial Least Squares (OPLS) model combining ^1^H-NMR and GC-MS data to identify characteristic metabolites in complex extracts. Both in metabolomics studies, and natural product chemistry, the reliable identification of marker metabolites usually requires laborious isolation and purification steps, which remains a bottleneck in many studies. Both ginger (GR) and processed ginger (PGR) are listed in the Japanese pharmacopeia. The plant of origin, the rhizome of *Zingiber officinale* Roscoe, is differently processed for these crude drugs. Notably, the quality of crude drugs is affected by genetic and environmental factors, making it difficult to maintain a certain quality standard. Therefore, characteristic markers for the quality control of GR and PGR are required. Metabolomic analysis using ^1^H-NMR was able to discriminate between GR and PGR, but there were unidentified signals that were difficult to distinguish based on NMR data alone. Therefore, we combined ^1^H-NMR and GC-MS analytical data to identify them by OPLS. As a result, α*r*-curcumene was found to be a useful marker for these identifications. This new approach enabled rapid identification of characteristic marker compounds and reduced the labor involved in the isolation process.

**Graphical abstract:**

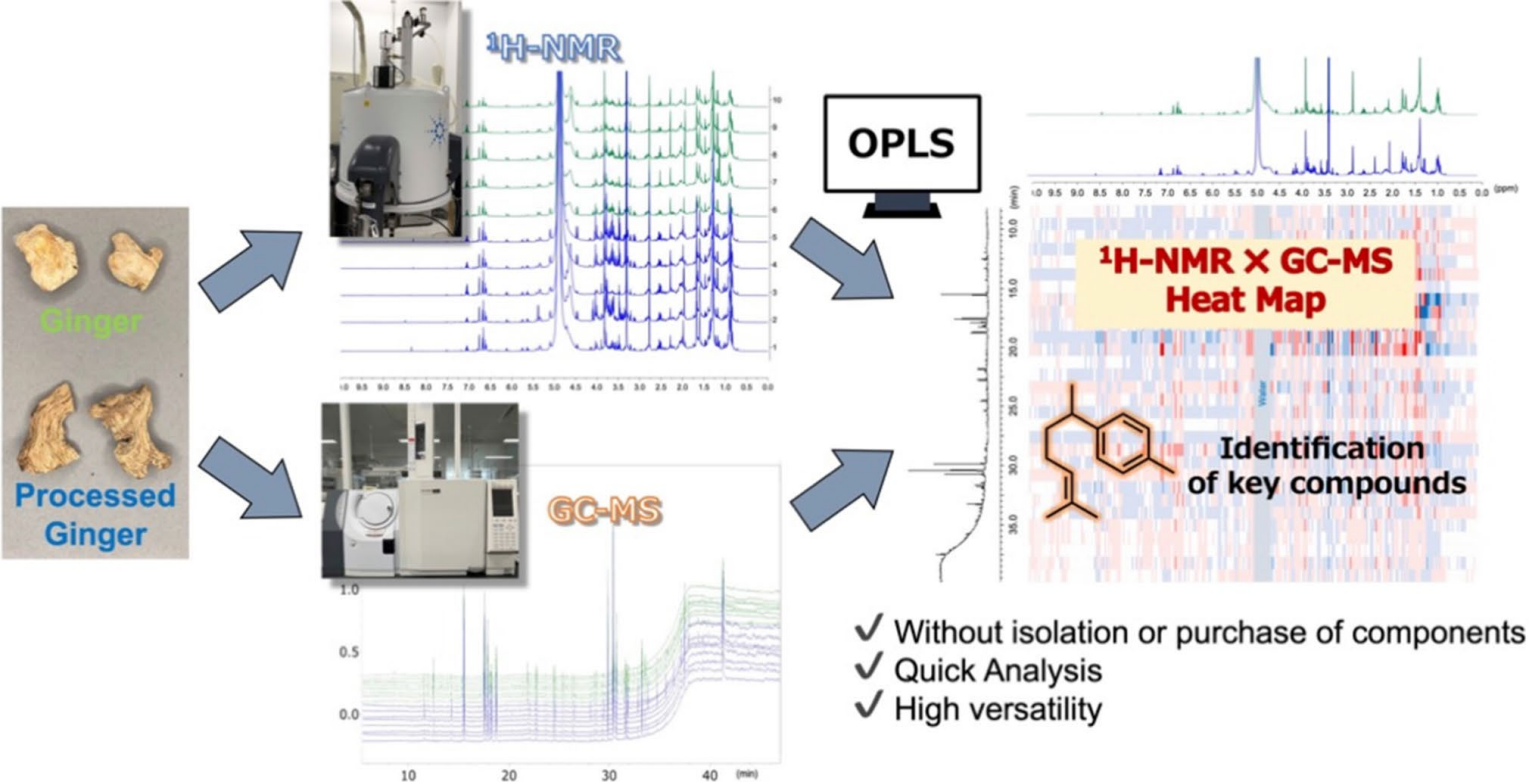

**Supplementary Information:**

The online version contains supplementary material available at 10.1007/s11418-024-01837-8.

## Introduction

Metabolomics has had a significant impact on many areas of research, including studies to understand disease pathogenesis, discovery of new drugs, assessment of crude drug quality and authenticity, and the identification of biomarkers [1, 2]. To date, metabolomic analyses have been performed on data matrices using many analytical instruments. These data acquisition methods can be divided into two types: separation and non-separation analyses [3]. Representative examples of separation analyses include LC-MS [4] and GC-MS [5]. These instruments are versatile because the sample preparation and elution systems (chromatographic separation) can be adapted to be extremely sensitive and provide rich structural information. However, the pre-purification step for separation analysis cannot detect all metabolites present in the crude drug. On the other hand, the “non-separation analysis type” has been reported for near-infrared (NIR) [6, 7], NMR [8, 9], and direct-injection MS [10, 11]. Non-separation analysis has been successfully applied in many areas, including the analysis of herbal medicines, due to its unique advantages, such as easy sample preparation and high robustness. NMR spectroscopy has the advantages of simultaneous detection of primary and secondary metabolites and specific structural characterization, and NMR-based metabolomics technology can solve the common problems of chromatographic approaches [12]. However, it is difficult to identify compounds using NMR-based chemometric approaches because one compound has multiple signals. The identification of marked compounds requires a standard purchased from a company or isolated. In general, the standard is expensive, and many compounds are not available for sale. In addition, isolating compounds requires considerable time and is difficult. Therefore, we developed an Orthogonal Partial Least Squares (OPLS) model combining ^1^H-NMR and GC-MS data, taking advantage of the “separation analysis type” and “non-separation analysis type” to facilitate reliable identification of metabolites from complex mixtures without individual isolation. Herein, this method was applied to identify a component that serves as a marker for the degree of rhizome processing in *Zingiber officinale* Roscoe, which is listed in Japanese pharmacopeia.

The rhizome of* Z. officinale* has long been used medicinally in Asian countries such as China, Korea, and Japan to treat colds, headaches, nausea, and gastrointestinal disorders [13–18]. Gingerols, a series of analogs with various unbranched alkyl chain lengths, activate the thermoreceptor Transient Receptor Potential Vanilloid 1 (TRPV1) and are the main pharmacologically active constituents in the rhizome of* Z*. *officinale *[4, 19]. The activity of [6]-shogaol against rat TRPV1 was approximately eightfold stronger than that of [6]-gingerol [20]. The rhizome of* Z*. *officinale* has a distinctively sweet and spicy aroma. The essential oil components of the *Z*. *officinale* rhizome, with its distinctive aroma, are composed of a variety of terpenoids [21, 22]. Essential oil components have antimicrobial activity, and the intensity of this activity depends on the constituents, such as α-zingiberene, α*r*-curcumene, and α-bisabolene [23]. In Japan, the rhizome of* Z*. *officinale* is processed to alter its function and enhance its efficacy. Based on the differences in processing, these crude drugs are classified by the Japanese Pharmacopoeia Eighteenth Edition (JP18) into two types: Ginger, GR (Shokyo in Japanese), which is dried only, and Processed Ginger, PGR (Kankyo in Japanese), which is passed through hot water or steamed [24]. The [6]-gingerol present in the rhizome of* Z*. *officinale* is partially dehydrated to [6]-shogaol and zingerone by steaming or boiling [25, 26]. Additionally, in JP18, two crude drugs were identified based on their [6]-gingerol and [6]-shogaol contents. Furthermore, the nature of GR is “pungent” and “warm”, while that of PGR is “strong pungent” and “hot”. This is because PGR contains more [6]-shogaol, which has a higher TRPV1 activation potential. In traditional Japanese Kampo medicine, Sohaku Asada, in his textbook”Kohoyakugi” [27] describes GR for mainly stopping vomit and curing phlegm and PGR for warming the inside of the body, stopping vomit and diarrhea, and treating cold abdomen and frequent urination and bowel movements at night. In other words, GR is used for patients with severe surface heat and vomiting, whereas PGR is used for patients with pain and other symptoms caused by the cold.

Quality assurance is important for ensuring the efficacy and safety of crude drugs, and a certain level of quality must be maintained [2]. However, JP18 does not refer to the specific manufacturing conditions for crude drugs of *Z*. *officinale* rhizome. Notably, the contents of [6]-gingerol and [6]-shogaol are affected by environmental factors such as developmental stage, harvest time, and geographic origin of the plant, making it difficult to guarantee a manufacturing quality. Therefore, multifaceted and comprehensive standardization through quality evaluation of GR and PGR is required.

In this study, we performed chemometric analysis using a complementary combination of ^1^H-NMR and GC-MS analytical data to generate heat maps of ginger rhizome extracts and estimate characteristic metabolites. The accuracy of our approach was validated through quantitative analysis using GC-FID. The method we devised greatly streamlines the isolation process and is applicable to a wide range of fields in metabolomics research and natural product chemistry.

## Result and discussion

### Identification of compounds in CD_3_OD extracts of GR and PGR by ^1^H-NMR

**Fig. 1:**
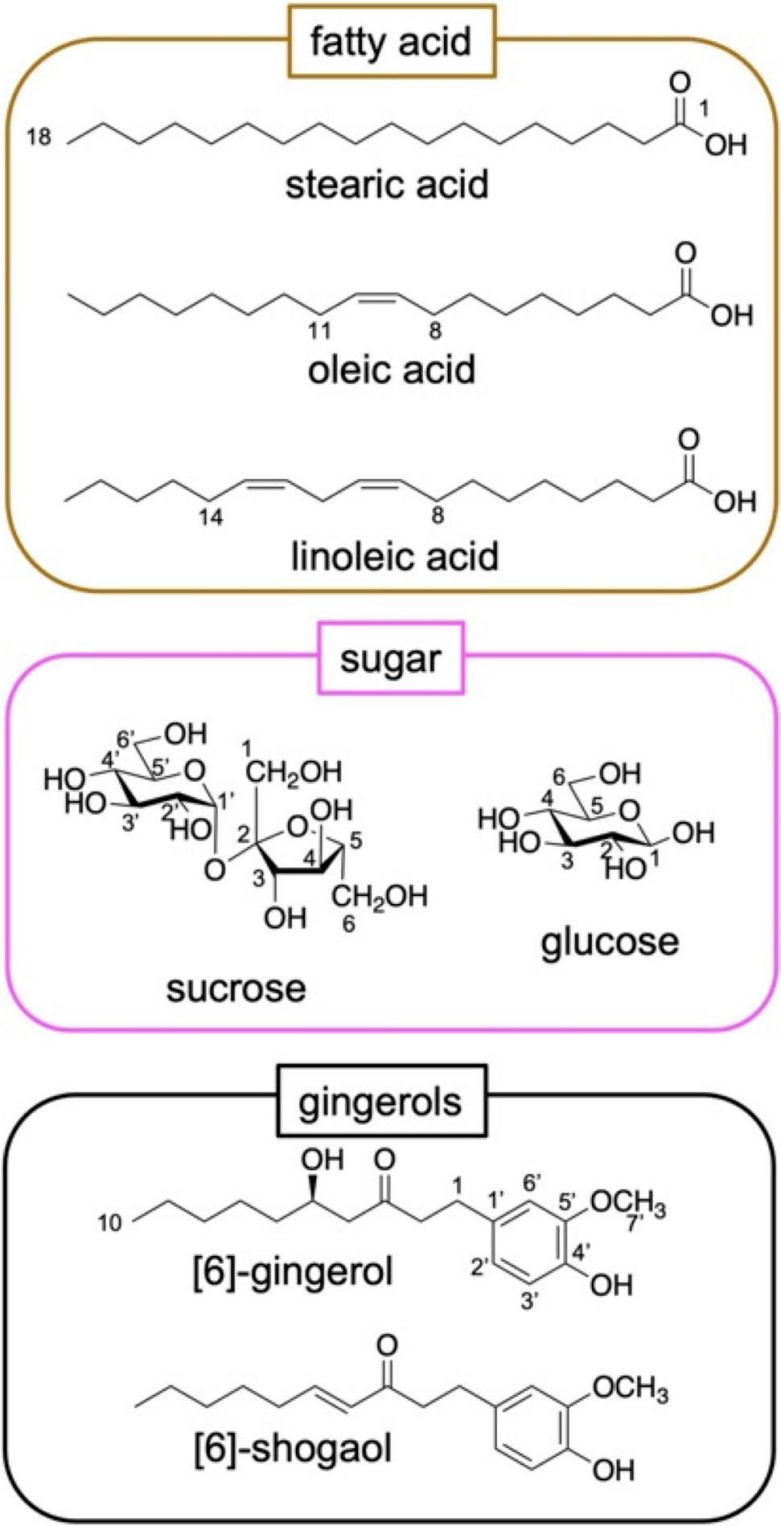
Structures of reference standard compounds

The ^1^H-NMR spectra of each sample extract were compared with the spectra of seven standards, as shown in Fig. 1, 2. The standards contained three fatty acids (FA): stearic acid (Ste), oleic acid (Ole), and linoleic acid (Lin); overwrapped sugars (SUG): sucrose (Suc) and glucose (Glu); and [6]-gingerol (6-Gin) and [6]-shogaol (6-Sho) standards of GR and PGR, as listed in JP18 (Table 1). The main SUG peaks were attributed to Suc (*δ* 5.35, 4.11–3.98, 3.85–3.56, 3.43–3.30) and Glu (*δ* 5.35, 4.11–3.98, 3.85–3.56, 3.43–3.30). In addition to Suc and Glu, overlapped signals presumably derived from various SUG were observed around *δ* 4.5–3.0. Ste, Ole, and Lin were observed at *δ* 2.27 (t, *J* = 7.4 Hz) and methylene groups at *δ* 1.31 (m) and *δ* 0.92–0.86 (m, H-18), which are common signals for C18 fatty acids. Olefin-derived signals for Ole and Lin were observed at *δ* 5.33 (m), *δ* 1.59 (m, H-3), and *δ* 2.02 (m, Ole H-8, 11 and Lin H-8, 14). The vanillyl group peaks of both compounds appeared characteristically at *δ* 6.78–6.59. The signal of H-4 of 6-Gin appeared specifically at *δ* 2.59–2.45. H-1and H-2 (4H integration signals) were observed as brs at *δ* 2.77, and the 6-Sho H-4 and H-5 signals were observed in the olefin region at *δ* 6.88 (dt, *J* = 15.9, 7.0 Hz) and 6.10 (dt, *J* = 15.9, 1.5 Hz).

**Fig. 2:**
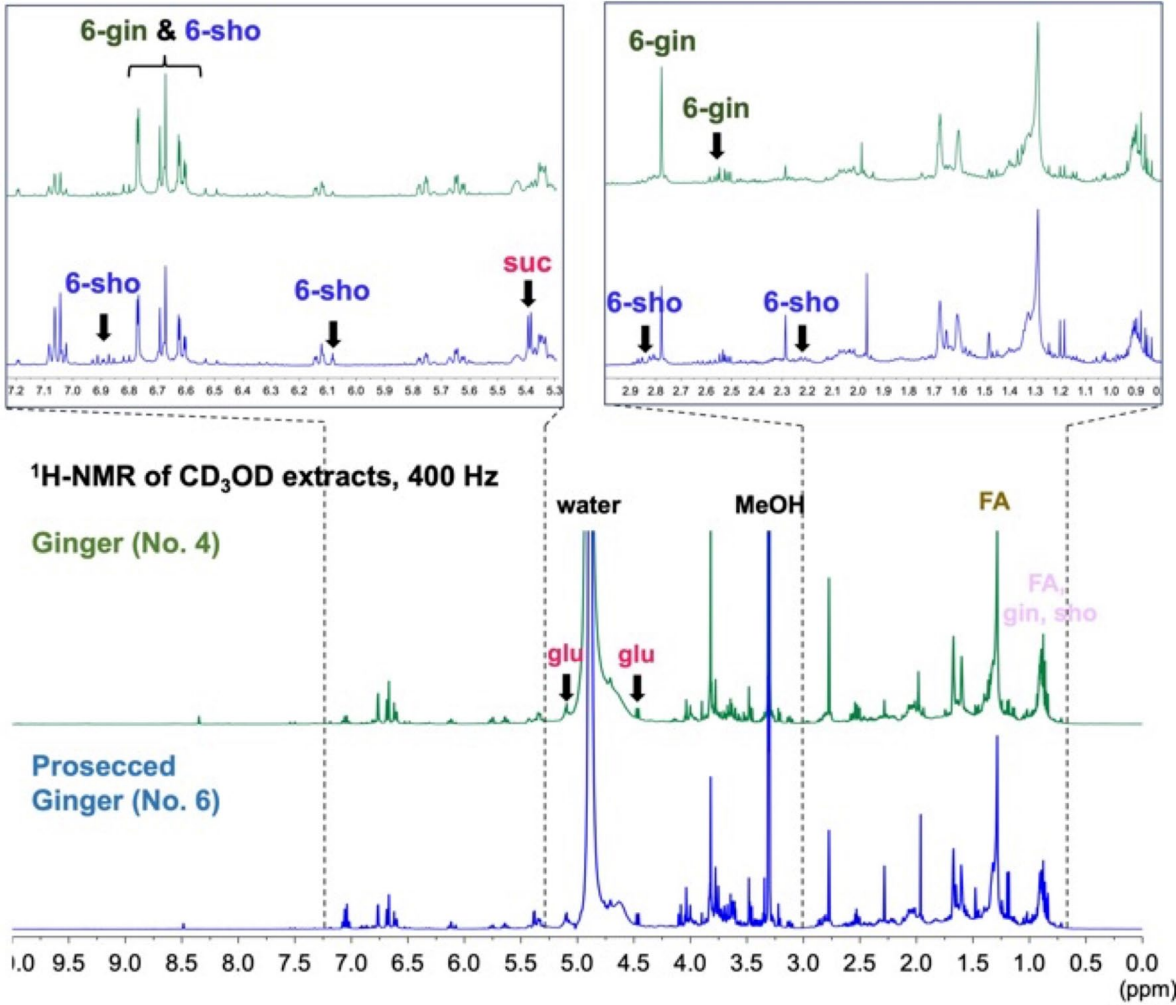
Representative ^1^H-NMR spectra of CD_3_OD extracts of GR (upper) and PGR (lower). Extended spectral regions (*δ* 0.70–3.0, 5.25–7.25) are shown on top of the figure

**Table 1:** Proton signals of reference compounds

Compound		Position	^1^H-NMR				Bucket
Stearic acid	Ste	2	2.27	t	*J* = 7.4 Hz	2H	2.30, 2.26
		3	1.64–1.54	m		2H	1.62, 1.58, 1.54
		4 ~ 17	1.22–1.38	m		26H	1.38, 1.34, 1.30, 1.26, 1.22
		18	0.89	brt		3H	0.94, 0.90, 0.86
Oleic acid	Ole	9, 10	5.34	m		2H	5.38, 5.34, 5.30
		2	2.27	t	*J* = 7.4 Hz	2H	2.30, 2.26
		8, 11	2.03	m		4H	2.06, 2.02, 1.98
		3	1.59	m		2H	1.62, 1.58, 1.54
		4 ~ 7, 12 ~ 17	1.41–1.21	m		20H	1.38, 1.34, 1.30, 1.26, 1.22
		18	0.89	brt		3H	0.94, 0.90, 0.86
Linoleic acid	Lin	9 ~ 13	5.40–5.27	m		4H	5.38, 5.34, 5.30, 5.26
		11	2.77	t	*J* = 6.1 Hz	2H	2.78, 2.74
		2	2.27	t	*J* = 7.4 Hz	2H	2.30, 2.26
		8, 14	2.06	m		4H	2.10, 2.06, 2.02
		3	1.59	m		2H	1.62, 1.58, 1.54
		4 ~ 7, 15 ~ 17	1.24–1.42	m		14H	1.42, 1.38, 1.34, 1.26
		18	0.9	brt		3H	0.94, 0.90, 0.86
Sucrose	Suc	1'	5.38	d	*J* = 3.8 Hz	1H	5.38
		1	3.63	d	*J* = 12.1 Hz	1H	3.62
			3.59	d	*J* = 12.1 Hz	1H	3.58
		3	4.09	d	*J* = 8.1 Hz	1H	4.10, 4.06
		4	4.01	dd	*J* = 8.1, 7.9 Hz	1H	4.02, 3.98
		3,5,6,5’,6’	3.84–3.67	m		7H	3.82, 3.78, 3.74, 3.70, 3.66
		2'	3.41	dd	*J* = 9.8, 3.8 Hz	1H	3.42, 3.38
		4'	3.37–3.34	m		1H	3.34
Glucose	Glu	α-1	5.09	d	*J* = 3.7 Hz	1H	5.10
		β-1	4.46	d	*J* = 7.8 Hz	1H	4.46
		α-2	3.80–3.74	m		1H	3.82, 3.78, 3.74
		α-3	3.7–3.63	m		1H	3.70, 3.66, 3.62
		α-4	3.34	dd	*J* = 3.7, 9.5 Hz	1H	3.34
		α-5	3.31			1H	–
		β-2	3.14–3.10	m		1H	3.14, 3.10
[6]-gingerol	6-Gin	6'	6.77	d	*J* = 1.9 Hz	1H	6.78, 6.74
		3'	6.68	d	*J* = 8.0 Hz	1H	6.70, 6.66
		2'	6.61	dd	*J* = 8.0 1.9 Hz	1H	6.62, 6.58
		5	3.99	m		1H	4.02, 3.98
		OCH_3_	3.82	s		3H	3.82
		1, 2	2.77	brs		4H	2.78
		4	2.59–2.45	m		2H	2.58, 2.54, 2.50, 2.46
		6	1.39	m		2H	1.42, 1.36
		7, 8, 9	1.35–1.21	m		6H	1.38, 1.34, 1.30, 1.26
		10	0.9	t	*J* = 6.7 Hz	3H	0.94, 0.90, 0.86
[6]-shogaol	6-Sho	5	6.89	dt	*J* = 15.9, 7.0 Hz	1H	6.94, 6.90, 6.86
		6'	6.77	d	*J* = 1.9 Hz	1H	6.78, 6.74
		3'	6.68	d	*J* = 8.0 Hz	1H	6.70, 6.66
		2'	6.61	dd	*J* = 8.0 1.9 Hz	1H	6.62, 6.58
		4	6.10	dt	*J* = 15.9, 1.5 Hz	1H	6.10, 6.06
		OCH_3_	3.82	s		3H	3.82
		1, 2	2.94–2.78	m		2H	2.90, 2.86, 2.82, 2.78
		6	2.22	m		2H	2.26, 2.22, 2.18
		7	1.46	m		2H	1.50, 1.46, 1.42
		8, 9	1.38–1.25	m		4H	1.34, 1.30, 1.26
		10	0.9	t	*J* = 6.7 Hz	3H	0.94, 0.90, 0.86

### PCA using ^1^H-NMR spectra of CD_3_OD extracts of GR and PGR

**Fig. 3:**
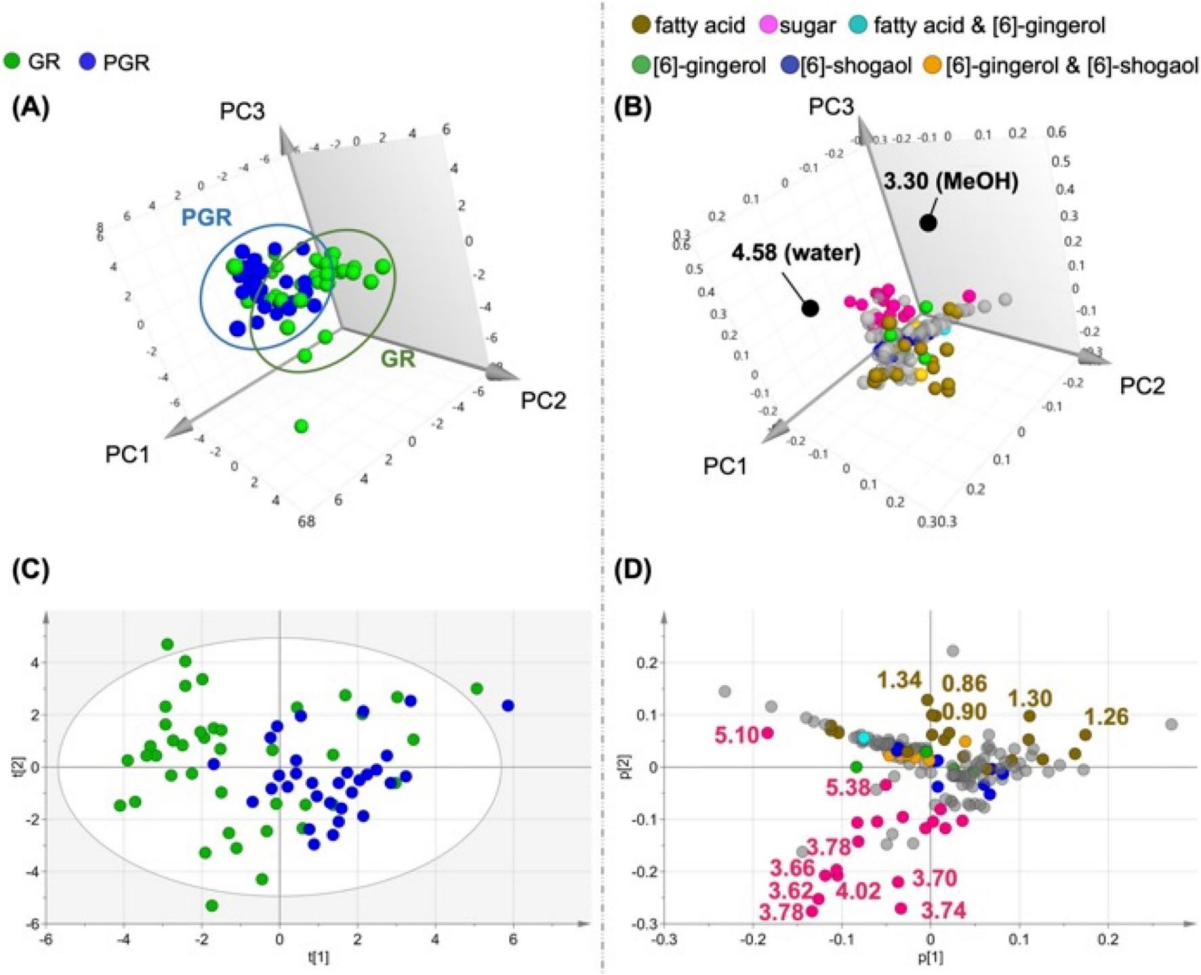
PCA using ^1^H-NMR spectrum extracted with CD_3_OD of GR and PGR. **A** 3D score plot of PC1, PC2 and PC3 scores. The variables are as follows—green; GR, blue; PGR. **B** 3D loading plot of PC1, PC2 and PC3 components. The variables are as follows—brown, fatty acids; pink, sugars; light blue, fatty acids and 6-gingerol; green, 6-gingerol; blue, 6-shogaol; and orange, 6-gingerol and 6-shogaol. **C** Score plot of PC1 and PC2 scores. The variables are as follows—green; GR, blue; PGR. **D** Loading plot for PC1 and PC2 components. The variables are as follows—brown, fatty acids; pink, sugars; light blue, fatty acids and 6-gingerol; green, 6-gingerol; blue, 6-shogaol; and orange, 6-gingerol and 6-shogaol

Principal component analysis (PCA), an unsupervised approach that can reflect pattern variation among samples, was used to examine changes in comprehensive composition due to the differences in processing GR and PGR. Using the Pareto scaling (Par) method, the contributions of PC1, PC2, and PC3 were 26.9%, 25.6%, and 12.3%, respectively (Fig. 3A). The buckets of both GR and PGR tended to be separated on the PC1 axis and distributed on the PC2 and PC3 axes. Analysis of the loading plot corresponding to PC3 revealed contributions from MeOH-derived *δ* 3.30 and water-derived *δ* 4.56 buckets (Fig. 3B). Therefore, PC3 might reflect the fullness of the crude drug extract. As PC3 discriminated between the differences in the amounts of compounds contained in the extracts, loading plots were analyzed, focusing on the chemical components contributing to PC1 and PC2 (Fig. 3C, D).

The pink buckets derived from the anomeric protons of Suc (*δ* 5.38) and SUG at *δ* 5.00–3.00 were spread over the third and fourth quadrants, while the brown buckets of the high-field peak around *δ* 1.34–1.26, derived from the methylene groups of FA, were spread over the first and second quadrants. These results suggest that the primary metabolites (FA and SUG) influenced the spread of PC2. OPLS-Discriminant Analysis (DA) was conducted because PCA did not clearly show peaks that could distinguish between GR and PGR.

### OPLS-DA using ^1^H-NMR spectra of CD_3_OD extracts

**Fig. 4:**
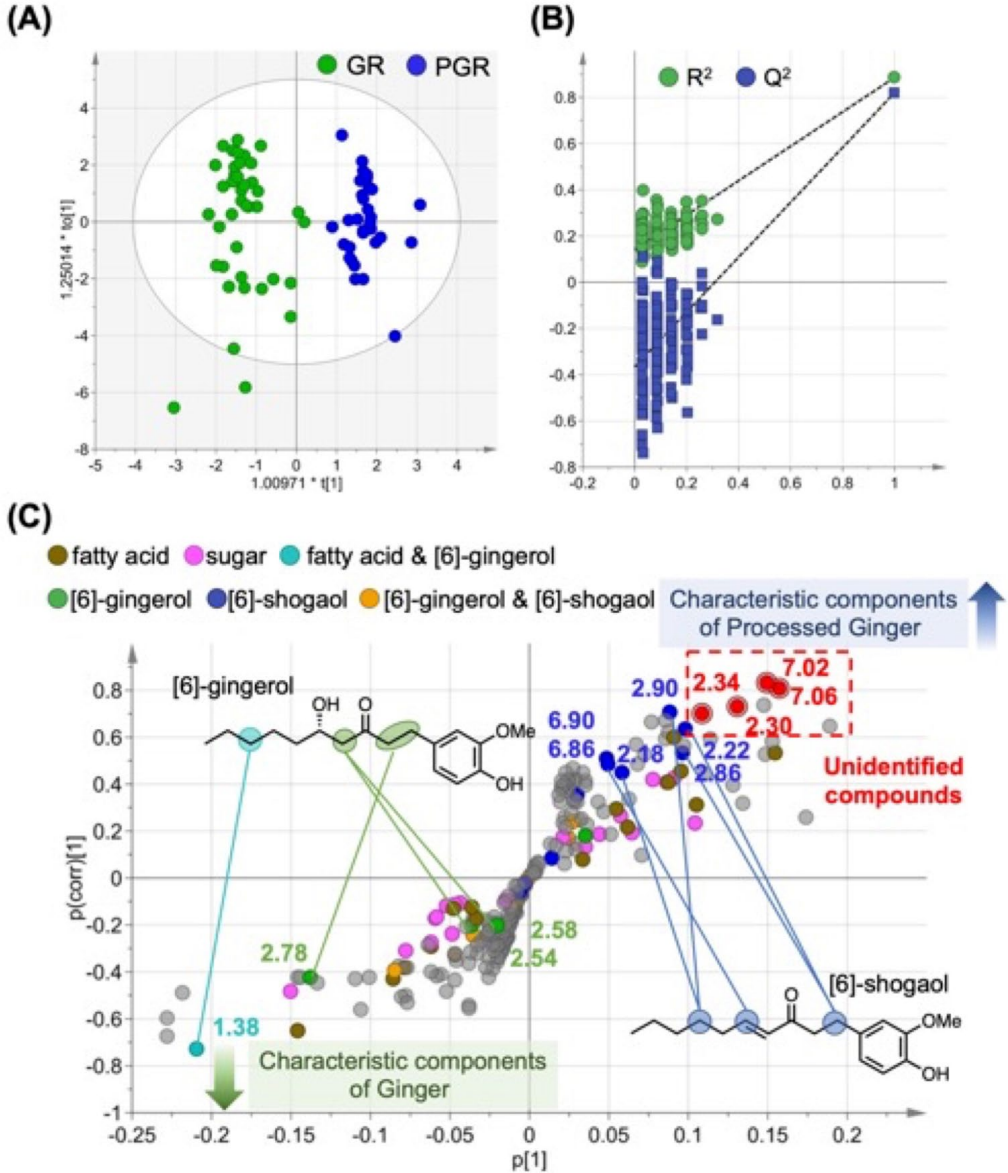
OPLS-DA using ^1^H-NMR of CD3OD extracts. **A** OPLS-DA score plot. **B** Statistical validation of the OPLS-DA analysis result by permutation tests. **C** S-plot of the OPLS-DA model. The variables in S-plots are as follows—brown, fatty acids; pink, sugars; light blue, fatty acids and 6-gingerol; green, 6-gingerol; blue, 6-shogaol; and orange, 6-gingerol and 6-shogaol. OPLS-DA,

OPLS-DA, classified into the GR and PGR groups, was implemented to search for the characteristic ^1^H-NMR signals of both crude drugs. OPLS-DA is a supervised approach with the advantage of determining the maximum separation between sample classes and identifying biomarkers. The model fit parameters were described using *R*^*2*^*Y* values for accuracy and *Q*^*2*^ values for confidence. A prediction model with *R*^*2*^*Y* > 0.65 and *Q*^*2*^ > 0.5 is considered suitable for quantitative prediction, and values close to 1.0 indicate a superior fit to the model [28]. The model fit parameters in this study were superior because of the 1 + 2 + 0 components, with *R*^*2*^*Y* = 0.886 and *Q*^*2*^ = 0.820. The Permutation test is used to validate the incidence of overfitting in a predictive model, and the model is valid if *R*^*2*^ and *Q*^*2*^ values are plotted on the X- and Y-axes and are less than 0.3 and 0.05, respectively [4, 29]. In this model, the results of 200 permutation tests are shown in Fig. 4B, with *R*^*2*^ = 0.142 and *Q*^*2*^ = -0.366, suggesting that it is a valid model.

The score plots showed a clear separation between GR and PGR (Fig. 4A). An S-plot consisting of the contribution on the X-axis and confidence on the Y-axis was used to search for the chemical shift characteristics of GR and PGR (Fig. 4C). The bucket characteristics of the PGR were distributed positively along the Y-axis, whereas the characteristics of the GR were distributed negatively. Characteristic buckets of PGR are shown on the positive X-axis of the S-plot, while those of GR are shown on the negative. Buckets with large |p(corr)| values were the characteristic signals for each crude drug.

Buckets derived from *δ* 1.35–1.25 for the methylene group of FA and *δ* 0.83–0.94 for the methyl group broad signal, as well as from *δ* 4.30–3.40 for SUG and anomeric protons, *δ* 5.38 for Suc and Glu (d, *J* = 3.8 Hz), and *δ* 5.10 (d, *J* = 3.8 Hz) were dispersed in both crude drugs. To identify the metabolite characteristics of both crude drugs, the analysis focused on the secondary metabolites present in the rhizomes of* Z*. *officinale*. 6-Gin and 6-Sho are known components of the rhizome of *Z. officinale* content; the bucket of 1.38 derived from the methylene groups of 6-Gin contribute to GR. On the other hand, buckets of 2.18, 2.22, 2.86, 2.90 derived from the methylene groups of 6-Sho and the bucket of 6.90 and 6.86 derived from olefine contributed to the PGR.

However, it was found that the buckets of 7.06, 7.02, 2.34, and 2.30 derived from the signals of the unidentified compounds contributed the most to PGR. The direct comparison with the ^1^H-NMR spectra of known compounds could not identify the buckets of 7.06, 7.02, 2.34, and 2.30. The signals contributing to the compounds were not identified by direct comparison with the ^1^H-NMR spectra. It was difficult to identify the compounds that produced the unassigned peak by analyzing the extract as a mixture utilizing only ^1^H-NMR.

Therefore, we decided to combine the data obtained from GC-MS, which facilitated compound identification, with the results of multivariate analysis of ^1^H-NMR.

### Identification of essential oil constituents in acetone extracts of GR and PGR by GC-MS

**Fig. 5:**
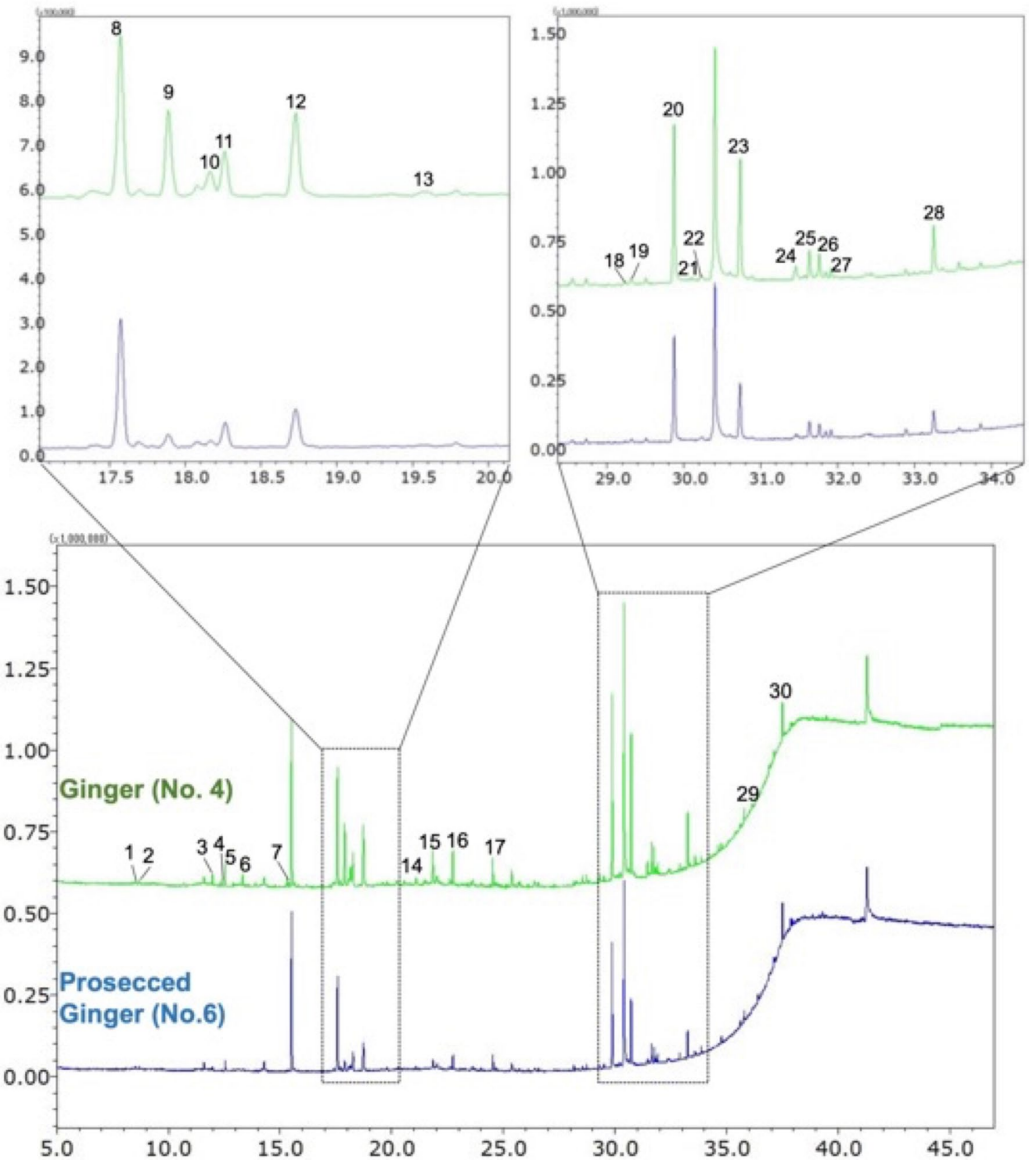
Comparison of GC-MS total ion current chromatograms of acetone extracts of GR and PGR, GR, ginger; PGR, processed ginger

Figure 5 shows the total ion current (TIC) chromatograms of acetone extracts of GR and PGR obtained using GC-MS. A total of 30 volatile compounds were identified in the quality control (QC) sample, including 13 terpenoids such as α*r*-curcumene and α-zingiberene, which are characteristic of the *Z*. *officinale* rhizome, and 12 gingerols, which are pungent compounds (Table 2).

**Table 2:** Volatile constituents found in hexane extracts from QC samples

No	Compounds	Rt (min)	RI
1	Octanal	8.67	1006
2	2,6-Dimethyl-nonane	8.96	1028
3	Borneol	11.97	1139
4	*α*-Terpineol	12.37	1174
5	Decanal	12.54	1207
6	Citral	13.60	1276
7	Copaene	15.39	1386
8	*αr*-Curcumene	17.57	1487
9	*α*-Zingiberene	17.89	1501
10	*α*-Farnesene	18.17	1511
11	*α*-Bisabolene	18.26	1515
12	Sesquiphellandrene	18.73	1531
13	Nerolidol	19.79	1569
14	Zingiberenol	21.10	1622
15	Zingerone	21.84	1658
16	Cedrenol	22.75	1704
17	Acorenone B	24.53	1817
18	[6]-Isoshogaol	29.24	2241
19	[6]-Paradol	29.32	2250
20	[6]-Shogaol	29.87	2312
21	Methyl-[6]-Shogaol	30.10	2337
22	[6]-Gingerdione	30.24	2354
23	[6]-Gingerol	30.74	2413
24	Diacetoxy-[6]-gingerdiol	31.46	2501
25	[8]-Shogaol	31.64	2526
26	Methyldiacetoxy-[6]-gingerdiol	31.77	2544
27	[10]-Shogaol	33.26	2747
28	[10]-Gingerdione	33.58	2793
29	Geranial acetal of [6]-gingerdiol	35.79	3116
30	*β*-Sitosterol	37.50	3394

### Orthogonal partial least squares (OPLS) combining ^1^H-NMR spectra and GC-MS chromatography

**Fig. 6:**
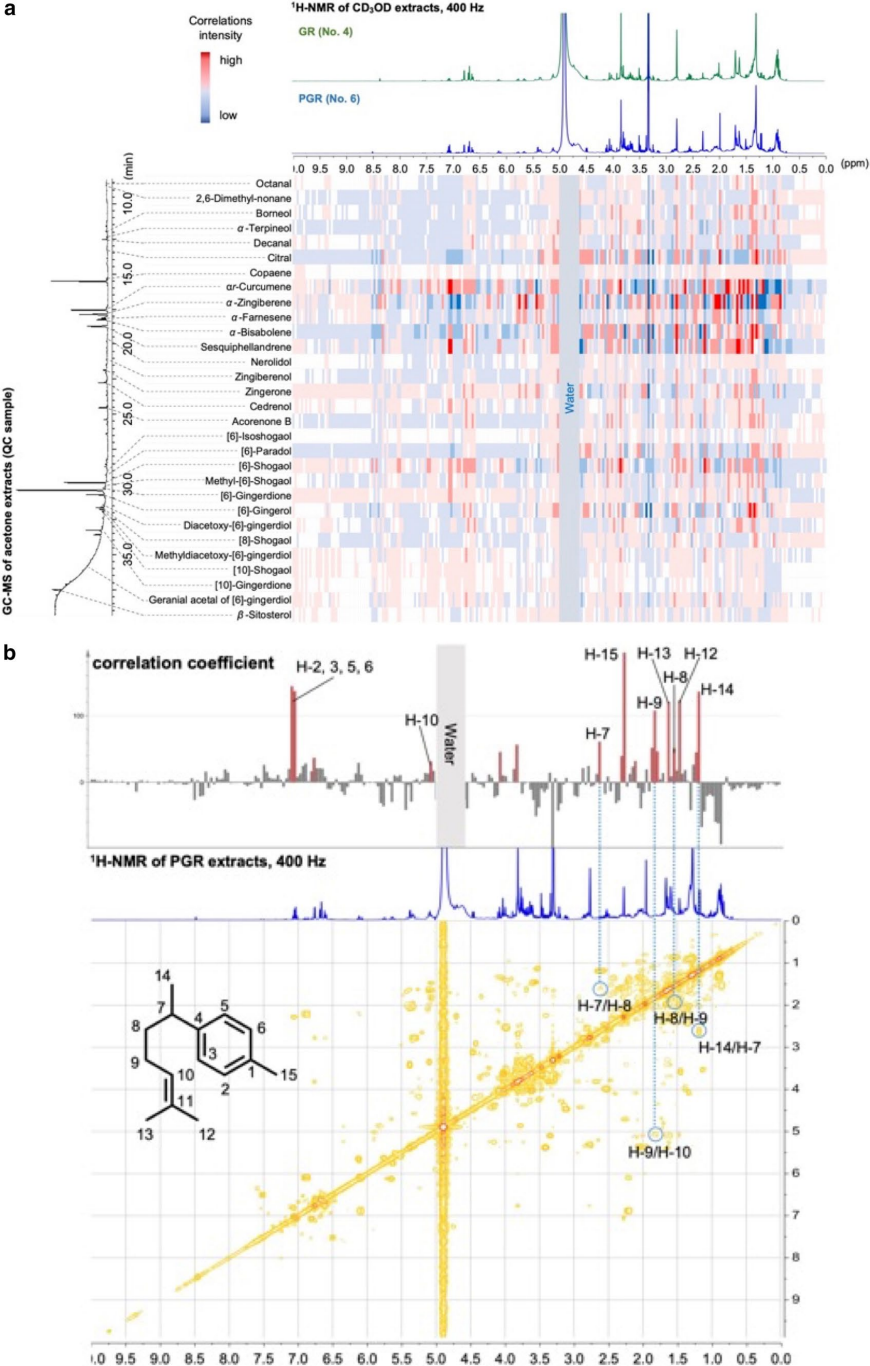
**A** Heatmap of correlation coefficient (r) among the profiling of ^1^H-NMR and GC-MS. The color scale indicates the correlation coefficient (r) values. **B** Identification of α*r*-curcumene by ^1^H-^1^H COSY of MeOH extracts of PGR using the correlation coefficient as an indicator

OPLS was performed to analyze the correlation between chemical features obtained by the different analytical techniques, ^1^H-NMR and GC-MS [30]. ^1^H-NMR spectral data (X variable) and TIC peak intensities of 30 compounds identified by GC-MS (Y variable) were analyzed to determine the correlation between GR and PGR. The correlation between the data obtained by ^1^H-NMR and GC-MS for the metabolites was calculated. The correlation coefficients between ^1^H-NMR and GC-MS obtained by OPLS are shown in Fig. 6 as a heat map. The color-coded scale from blue to red represents the change from lower to higher correlations between the data obtained by ^1^H-NMR and GC-MS for the metabolites. The parameters were *R*^*2*^*Y* = 0.485 and *Q*^*2*^ = 0.366, with low precision and confidence, for analyzing both the X and Y variables on a Pareto scale.

The low precision and confidence were thought to be induced by variations in ^1^H-NMR signal intensity owing to differences in the content of various crude drug compounds. In addition, the structures of the gingerols are similar to those of FA, with buckets at *δ* 0.89–1.35 and *δ* 3.01–2.89 as overlapping signals and having a large number of protons, resulting in complex couplings with adjacent protons and few single signals. Therefore, the correlation between ^1^H-NMR and GC-MS for the gingerols was moderate. In contrast, sesquiterpenes contain multiple methyl groups, which result in a singlet signal with relatively large integrated values in the ^1^H-NMR spectrum. Furthermore, the ^1^H-NMR signals of sesquiterpenes had different chemical shifts from those of SUG and FA, owing to the presence of aromatic rings and olefins. In contrast to gingerols, sesquiterpenes exhibited a strong positive correlation between ^1^H-NMR and GC-MS.

### Confirmation of the unidentified compounds based on the ^1^H-NMR experiment

The characteristic unidentified buckets of 7.06, 7.02, 2.34, and 2.30 in the CD_3_OD extract of PGR were revealed by OPLS-DA to show a strong positive correlation with the peak with a retention time of 17.57 min on GC-MS, which was α*r*-curcumene. (Fig. 6A). Therefore, to confirm that the buckets of 7.06, 7.02, 2.34, and 2.30 were derived from *αr*-curcumene, the correlation intensities of the ^1^H-NMR-*αr*-curcumene of the CD_3_OD extract of the PGR extract were used as indicators for CD_3_OD. Figure 6B shows the correlation coefficients between each bucket obtained by ^1^H-NMR and *αr*-curucumene identified by GC-MS (high correlation coefficients are highlighted in red), and the derived ^1^H-^1^H COSY signals are shown. A correlation between H-14/H-7/H-8/H9/H-10 of the PGR CD_3_OD extracts and HMBC of H-15 to C-6, 2, H-12, and H-13 to C-10 was observed by ^1^H-^1^H COSY (Fig. S1-S4), and signals at *δ* 7.06, 7.02, 2.34, 2.30 were H-2, 3, 5, 6, and 15 of *αr*-curcumene. Upon isolation, the peak at 17.57 min on GC-MS was confirmed to be *αr*-curcumene. Resonance overlaps and complications hinder data interpretation and unambiguous metabolite identification, thereby limiting the application of NMR. However, OPLS combining ^1^H-NMR and GC-MS was able to identify signals of *αr*-curcumene in complex mixtures that could not be identified even by 2D-NMR and had a pivotal role in metabolite identification. Previously, *αr*-curcumene was not detected in the fresh rhizome of* Z*. *officinale* by GC-MS analysis, as reported by Yoshikawa et al. [31]. On the other hand,* αr*-curcumene is known to be formed by oxidation of unstable *α*-zingiberene during the processing of turmeric rhizomes [32, 33]. It is thought that α*r*-curcumene, the characteristic component of PGR found in this study, is increased by the processing of the rhizome of* Z*. *officinale*. Therefore, we confirmed the change from *α*-zingiberene to *αr*-curcumene by processing *Z. officinale* rhizomes.

### Determination of α-zingiberene/α*r*-curcumene by GC-FID

**Fig. 7:**
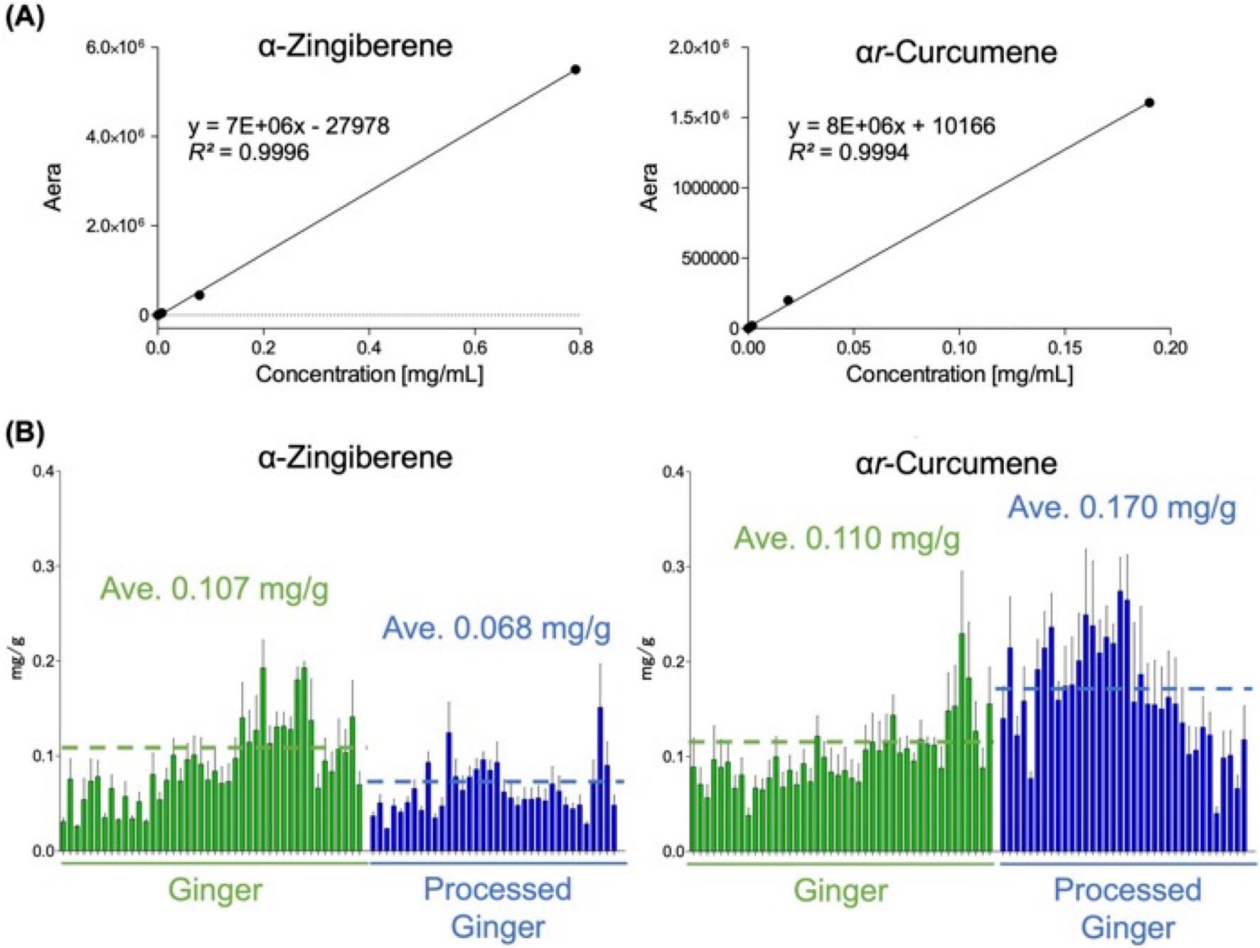
**A** Calibration curves of the corresponding *α*-zingiberene and *αr*-curcumene for the quantitative analysis. **B** Comparison of contents of *α*-zingiberene and *αr*-curcumene between GR and PGR

To support our predictive data, it was required quantification using GC-FID of *α*-zingiberene and *αr*-curcumene, after both components were isolated from *Z. officinale* rhizomes for use in the calibration curve. The structures were determined based on a careful comparison of the NMR data to those in the literature, and their ^1^H-NMR chemical shifts are shown in Table 3 [34–36]. As shown in Fig. 7A, the precision (*R*^*2*^) is very close to 1, and the y-axis intercept (b) is close to 0, indicating that the standard curve has excellent quantitative performance. Eighty GR and PGR samples were subjected to quantitative analyses using these calibration curves. The *α*-zingiberene content was 1.19 mg/g for GR and 0.85 mg/g for PGR, and *αr*-curcumene was 0.65 mg/g for GR and 1.30 mg/g for PGR (Fig. 7B). Notably, the OPLS-DA results were supported by the GC-FID quantification. However, in a few PGR samples, a lack of processing could be caused it to observe only slightly to change from *α*-zingiberene to *α*r-curcumene.

**Table 3:** ^1^H-NMR (400 MHz) and ^13^C-NMR (100 MHz) data of α-zingiberene and α*r-*curcumene

Compound	position	*δ*_C_	*δ*_H_					Bucket
*α*-Zingiberene	1	132.1	–	–				
	2	131.8	5.62	dd	*J* = 9.9, 3.0 Hz	1H		5.62
	3	26.9	2.07–1.88	m		1H		2.06, 2.02, 1.98, 1.94
	4	39.4	2.26–2.17	m		1H		2.22, 2.18, 2.14
	5	128.9	5.77	m		1H		5.78, 5.74
	6	121.2	5.42	br		1H		5.46, 5.42
	7	37.2	1.58–1.48	m		1H		1.58, 1.54, 1.50
	8	35.4	1.46–1.46	m		1H		1.46, 1.42, 1.38
			1.22–1.12	m		1H		1.22, 1.18, 1.14
	9	25.5	2.07–1.97	m		1H		2.06, 2.02, 1.98
	10	125.8	5.10	m		1H		5.14, 5.10, 5.06
	11	130.8	–	–				
	12	17.7	1.60	s		3H		1.62, 1.58
	13	25.9	1.68	m		3H		1.70, 1.66
	14	16.9	0.88	d	*J* = 6.9 Hz	3H		0.90, 0.86
	15	21.3	1.68	m		3H		1.70, 1.66
*αr*-Curcuene	1	134.8	–	–				
	2, 6	128.4	7.07	d	*J* = 8.3 Hz	2H	7.10, 7.06	
	3, 5	126.5	7.02	d	*J* = 8.3 Hz	2H	7.06, 7.02	
	4	144.2	–	–				
	7	38.9	2.62	sext	*J* = 7.0, 14.9 Hz	1H	2.66, 2.62, 2.58	
	8	38.2	1.57	m		2H	1.58, 1.54	
	9	25.8	1.90–1.78	m		2H	1.86, 1.82	
	10	124.3	5.08	m		1H	5.10, 5.06	
	11	130.7	–	–				
	12	16.3	1.48	brs		3H	1.50, 1.46	
	13	24.5	1.65	d	*J* = 1.1 Hz	3H	1.66, 1.62	
	14	21.6	1.19	d	*J* = 6.9 Hz	3H	1.18	
	15	19.6	2.29	s		3H	2.34, 2.30

**Fig. 8:**
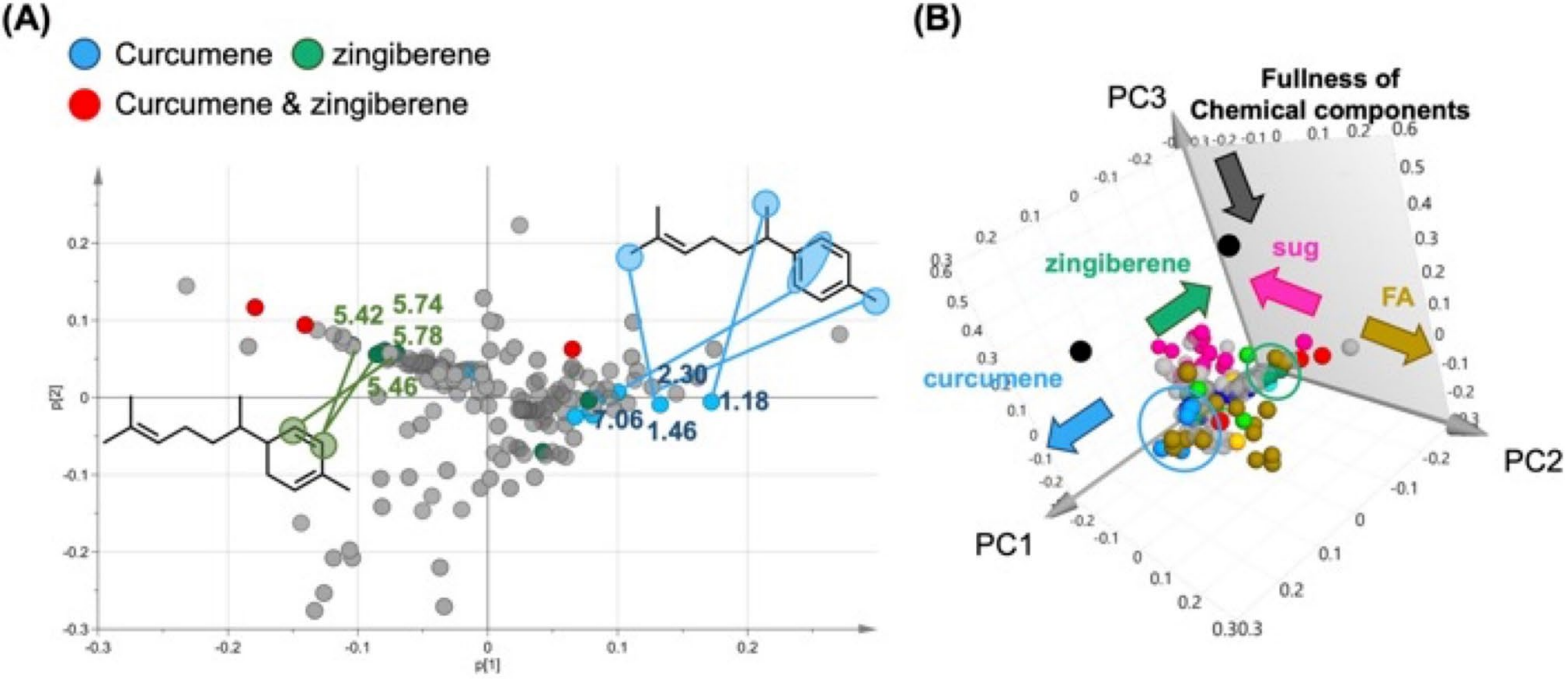
Loading plot after substitution of standard compounds for PC1, PC2, and PC3 constituents derived from PCA using ^1^H-NMR spectra of CD_3_OD extracts

Substitution of the signal identified as *αr*-curcumene into the PCA loading was consistent with the bucket contributing to the PC1 axis (Fig. 8). That is, the positive PC1 axis (quadrants 1 and 2) was dominated by *α*-zingiberene-derived signals while *α*r-curcumene-derived signals dominated the negative PC1 axis (quadrants 3 and 4). The score plot modeled in this study comprehensively reflected the component content, primary metabolites (sugars and fatty acids) and secondary metabolites (essential oils), and the PCA, OPLS-DA, and OPLS results were consistent, suggesting that these compounds are characteristic of GR and PGR.

## Conclusion

In this study, OPLS based on a complementary combination of ^1^H-NMR and GC-MS analytical data allowed us to identify *αr*-curcumene as a characteristic constituent of the PGR extract without the need for laborious fractionation operations. Different analytical instruments detect various compounds and produce diverse analytical results. Comprehensive statistical analysis of results obtained from different analytical methods can enhance the sensitivity and accuracy of analytical findings. In this study, GC-MS and ^1^H-NMR data were integrated to ensure the reliability of the complementary multivariate analysis. The method developed in this study greatly facilitates the identification of known and unknown metabolites from complex mixtures with comparatively little practical effort. Although the samples were measured at 64 scans, with recently improved NMR analytical techniques, this method can be extended to the identification of trace and unknown components by performing highly sensitive measurements. Furthermore, by using LC-MS as an alternative to GC-MS, this method can be applied for the identification of non-volatile components, which was not the focus of this study. In addition, this method is highly versatile because it can be applied not only to crude drugs but also to a wide range of natural products, including in the food industry. In addition, enhancing the robustness of this approach could enable the estimation of metabolites in complex mixtures to be estimated without the need for separate isolation.

*αr*-Curcumene is expected to be a new indicator component involved in assessing the quality and degree of processing of *Z. officinale* rhizome. The PCA of the CD_3_OD extract using ^1^H-NMR spectra showed no clear separation between the two on the score plot; however, there was a trend toward separation. OPLS-DA using ^1^H-NMR spectra was performed to identify the characteristic compounds of GR and PGR. The score plot showed the separation of each crude drug, with 6-Gin and 6-Sho, which are characteristic of *Z. officinale* rhizome, contributing only slightly to the separation. However, some compounds contributed more to the separation. To identify the compounds that contributed to the separation, correlation analysis was performed using the data obtained by GC-MS as the explanatory variables. The approach used in this study significantly shortened the isolation process and identified compounds characteristic of GR and PGR. *αr*-Curcumene was found to change from α-gingiberene by processing. These compounds could be useful as quality control markers and could signal the degree of process.

## Materials and methods

### Plant material

The details of the GR and PGR used in this study are presented in Table 4. GR and PGR samples were provided by the National Institutes of Biomedical Innovation, Health, and Nutrition (Ibaraki, Japan) and Kitasato University Oriental Medicine Research Center (Tokyo, Japan).

**Table 4:** Details of the crude drug samples used in this study

No	Drug name	Collection year	Producing area	Code No
1	Ginger	2006	Yunnan	NIB-0075
2		2008		NIB-0039
3		2008		NIB-0060
4		2008		NIB-0091
5		2009		NIB-0169
6		2010		NIB-0055
7		2010		NIB-0110
8		2010		NIB-0147
9		2010		NIB-0179
10		Unknown	Unknown	w160048002
11				90610
12				I6Q0227
13				US262108
14				08H0227
15				581001
16				240498
17				310198
18				15037721
19				16016521
20				13023581
21			Sichuan	17030821
22				19039651
23				20001711
24				20001771
25		2002		20050871
26		2002		21005861
27		2002		21027451
28		2003		21056291
29		2003		22013571
30		2003		22026821
31		2004		22030411
32		2004		22046221
33		2004		23014071
34		2005		24025291
35		2006		24026621
36		2006		24029251
37		2006		25021331
38		2012		E37041
39		2013		H12461
40		2014		J05851
41		2016	Unknown	K30901
101	Processed Ginger	2012	Guangxi Zhuang Autonomous Region	NIB-1115
102		2013		NIB-0787
103		2013		NIB-0788
104		2013		NIB-0789
105		2013	Yunnan	NIB-0801
106		2013	Guangxi Zhuang Autonomous Region	NIB-0802
107		Unknown	Unknown	24110002
108		Unknown		I5F0113
109		Unknown		40710
110		Unknown		2106
111		Unknown		9261056
112		2003	Yunnan	402124
113		2004		452123
114		2006		US312517
115		2009		90G0113
116		2010		O2H0113
117		2011		OBM0013
118		2012		B3L0113
119		2013		C3P0113
120		2014		D1F0113
121		2014		D5k0113
122		2015		E480113
123		2015		D0D0113
124		2016		F320113
125		2016		F610113
126		2017		G330113
127		2017	Guangxi Zhuang Autonomous Region	G4G0113
128		2017		G8M0113
129		2018		H7C0113
130		2018		H1J0113

### General equipment and chemicals

Samples were powdered using a high-speed vibration sample mill (CMT T1-100; CMT, Tokyo, Japan). A precise Mettler Toledo XS105 dual-range analytical balance was used to prepare the extracts for analysis. Sonication was performed using an Ultrasonic Cleaner (US-109; SND, Tokyo, Japan). KUBOTA 3740 (Kubota, Tokyo, Japan) and CVE-3000 (EYELA) were used for centrifugation. NMR spectra were obtained using an Agilent Technologies 400-MR (Agilent Technologies, Santa Clara, CA, USA) with ^1^H-NMR at 400 MHz in CD_3_OD (Fujifilm Wako Pure Chemical Co., Osaka, Japan). The chemical shifts are expressed in ppm downfield from the internal solvent peaks for CD_3_OD (*δ* 3.31 for ^1^H-NMR), and *J* values were measured in Hertz. GC-MS analyses were performed on a Shimadzu-2010 plus gas chromatograph equipped with a split/splitless injector coupled with a QP2010 Ultra MS detector and a SHIMADZU AOC-20i autoinjector. The column used for the application was RESTEK Rtx®-5MS (30 m × 0.25 mm ID × 0.25 μm film thickness), and helium (99.9%) was used as a carrier gas (1.5 mL/min). For quantitative analysis of *α*-zingiberene and *αr*-curcumene, the extracts were injected into a SHIMADZU GC-2014 and SHIMADZU AOC-20i autoinjector fitted with an FID detector. Semi-preparative HPLC was performed on a Jasco UV 2075 Plus detector coupled with a Jasco LC-Net 11/ADC valve, Jasco PU-2080 pump, and YMC-Actus Triart C18 reversed-phase (RP) analytical column (150 mm × 20 mm).

### Reagents

The 6-Gin and 6-Sho standards, fatty acids, and sugars were obtained from Fujifilm Wako Pure Chemical Co. (Osaka, Japan). The signals in the ^1^H-NMR spectra of the GR and PGR extracts were assigned to individual metabolites based on a thorough analysis of the 2D-NMR spectra and spiking experiments.

### Sample preparation for conventional NMR‐based metabolomics

Crude drug samples were powdered without pretreatment, and the powder was stored in a sealed airtight container until analysis. 1 ml of CD_3_OD was added to 100 mg of powdered GR and PGR. These powders were extracted by ultrasonication at room temperature for 1 h. After centrifugation (3000 rpm, 5 min), the supernatant was collected and filtered with a 0.45 μm filter. The filtrate was then transferred into 3-mm standard NMR tubes [8].

### NMR measurement

The 1D ^1^H- and ^13^C-NMR spectra were measured at 400 and 100 MHz, respectively, at a temperature of 298 K using a 45° excitation pulse (Bruker Pulprog: zg). The probe was frequency-tuned and impedance-matched before each acquisition. For each sample, 64 scans and four dummy scans were recorded using the following parameters: a spectral width of 16 ppm, relaxation delay of 3.0 s, and receiver gain of 256. The 2D ^1^H-^1^H COSY spectra were acquired with a 1.0 s relaxation delay, 6410.3 Hz spectral width in both dimensions. The number of scans per t_1_ increment was set to 64. The spectra were collected with 128 complex t_1_ and 512 complex t_2_ points for a measurement time of 11 h (digital resolution; 50.1 Hz).

### GC-MS measurement

Following the same procedure as for the preparation of metabolomics samples using NMR, 10 mg of Ginger or Processed Ginger powder was extracted with acetone containing 0.01 µl of ethyl decanoate (internal standard, IS) [37]. QC samples were obtained by mixing equal amounts of the prepared sample extract. The injection volume was 1 μL with a split ratio of 1:1. Ions were generated by electron ionization (EI). Spectra were recorded at 1666 u/s (check value) over the mass range *m/z* 45 − 500. The column temperature was maintained at 60 °C for 5 min, and then ramped at 10 °C/min up to 150 °C and maintained for 5 min, then at 10 °C/min up to 330 °C, and maintained for 10 min. The total run time was 47.0 min at a constant flow rate of 0.89 ml/min. The injector temperature was 250 °C, and the detector temperature was 200 °C.

### Identification of volatile compounds

A GC-Analyzer (MsMetrix, Maarssen, Netherlands) was used to identify the metabolites in the QC samples by matching the MS spectrum to the standard spectrum in the National Institute of Standards and Technology (NIST) 2020 Mass Spectra Database, as previously described [21–23, 38, 39], and confirmed by comparing the linear retention index (RI) calculated relative to (C_9_-C_40_) *n*-alkanes (GL Sciences, Tokyo, Japan).

### Preparation of data matrix and multivariate analysis

The NMR spectra were imported into ALICE2 for metabolome analysis (JEOL, Tokyo, Japan) to obtain a data matrix using the total integral mode. Each NMR spectrum was bucketed by integrating regions having an equal bin size of 0.04 ppm over a range of δ 0.00 to 10.00 ppm. Then, the median value of bines was used as the bucket value. The spectra were referenced to CH_3_OH at *δ* 3.30. The integral values of the signals of water (*δ* 5.00–4.60) were removed from data matrices of ^1^H-NMR spectra.

Data obtained from GC-MS were analyzed and preprocessed, including spectral deconvolution and retention time alignment, using the software program MsXelerator (MsMetrix, Maarssen, Netherlands). The original matrices obtained were standardized using the IS.

SIMCA-P (Version 14.1, Umetrics, Umea, Sweden) was used for the multivariate analysis of the normalized data matrices. To search for characteristic metabolites, PCA, OPLS-DA, and OPLS were performed on GR and PGR samples. The OPLS model was set up with the integrated value for 240 buckets of ^1^H-NMR as the *X* variable and the standardized peak areas of the 30 peaks identified by GC-MS analysis as the *Y* variable.

### Isolation of α-zingiberene and αr-curcumene

Ginger (Lot: N610227) was purchased from UCHIDAWAKANYAKU, Ltd. (Tokyo, Japan). Ginger (300 g) was powdered using a high-speed vibrating sample mill and extracted with hexane (3 L) in an ultrasonic bath for 2 h. The extract was passed through vacuum liquid chromatography (VLC) using silica gel and evaporated (1.37 g). The residue was separated using reverse-phase (RP) semi-preparative HPLC (MeOH:H_2_O = 95:5, 16 ml/min) to obtain *r*-curcumene (25.3 mg) and a mixture of *α*-zingiberene. The mixture was further purified using RP semi-preparative HPLC (MeOH:H_2_O = 9:1, 16 ml/min) to obtain *α*-zingiberene (13.4 mg).

### Quantitative analysis of α-zingiberene and α*r*-curcumene content in GR and PGR

*α*-Zingiberene and *αr*-curcumene in GR and PGR were quantified by using GC-FID in conditions similar to that in the GC-MS analysis. 10 mg of the sample (*n* = 3) was extracted by ultrasound with 1 mL of hexane for 60 min, followed by centrifugation and filtration. *α*-Zingiberene and *αr*-curcumene were dissolved in hexane to a concentration of 1 mg/ml and then diluted 10, 100, 1000, 10000, and 10000 times with hexane and filtered. 1 µl of these solutions were injected into the GC-FID. A calibration curve was prepared to analyze by the GC-FID system.

## Supplementary Information

Below is the link to the electronic supplementary material.

Supplementary file1 (PDF 1333 KB)
