# Correction: Development of a determination method for quality control markers utilizing metabolic profiling and its application on processed *Zingiber officinale* Roscoe rhizome

**DOI:** 10.1007/s11418-025-01900-y

**Published:** 2025-03-25

**Authors:** Tomohisa Kanai, Tatsuya Shirahata, Shunsuke Nakamori, Yota Koizumi, Eiichi Kodaira, Noriko Sato, Hiroyuki Fuchino, Noriaki Kawano, Nobuo Kawahara, Takayuki Hoshino, Kayo Yoshimatsu, Yoshinori Kobayashi

**Affiliations:** 1 School of Pharmacy, Kitasato University, 5–9–1 Shirokane, Minato-Ku, Tokyo, 108–8641 Japan; 2 Oriental Medicine Therapy Center, Kitasato Institute Hospital, Kitasato University, 5–9–1 Shirokane, Minato-Ku, Tokyo, 108–8641 Japan; 3 Research Center for Medicinal Plant Resources, National Institutes of Biomedical Innovation, Health and Nutrition, 1–2 Hachimandai, Tsukuba, Ibaraki 305–0843 Japan; 4 The Kochi Prefectural Makino Botanical Garden, Godaisan, Kochi, 781–8125 Japan

**Correction: Journal of Natural Medicines (2024) 78:952–969** 10.1007/s11418-024-01837-8

The article “Development of a determination method for quality control markers utilizing metabolic profiling and its application on processed *Zingiber officinale* Roscoe rhizome”, written by Tomohisa Kanai, Tatsuya Shirahata, Shunsuke Nakamori, Yota Koizumi, Eiichi Kodaira, Noriko Sato, Hiroyuki Fuchino, Noriaki Kawano, Nobuo Kawahara, Takayuki Hoshino, Kayo Yoshimatsu and Yoshinori Kobayashi, was originally published under exclusive license to The Author(s) under exclusive licence to The Japanese Society of Pharmacognosy. As a result of the subsequent decision to publish the article under the open access model, the article’s copyright notice was changed on 6 March 2025 to © The Author(s) 2025 and the article is now distributed under a Creative Commons Attribution 4.0 International License, which permits use, sharing, adaptation, distribution and reproduction in any medium or format, as long as you give appropriate credit to the original author(s) and the source, provide a link to the Creative Commons licence, and indicate if changes were made. The images or other third party material in this article are included in the article’s Creative Commons licence, unless indicated otherwise in a credit line to the material. If material is not included in the article’s Creative Commons licence and your intended use is not permitted by statutory regulation or exceeds the permitted use, you will need to obtain permission directly from the copyright holder. To view a copy of this licence, visit http://creativecommons.org/licenses/by/4.0.

The original article has been corrected.

