# Supplementary material for: Development of a determination method for quality control markers utilizing metabolic profiling and its application on processed Zingiber officinale Roscoe rhizome
Source: J Nat Med. 2024 Aug 3;78(4):952–69. doi: 10.1007/s11418-024-01837-8 (PMC11937189; doi:10.1007/s11418-024-01837-8)
Supplement: Supplementary file 1 — Supplementary file1 (PDF 1333 KB) [file 11418_2024_1837_MOESM1_ESM.pdf]

Electronic supplementary material

Development of a determination method for quality control markers utilizing metabolic profiling and its application on processed *Zingiber officinale* Roscoe rhizome

Tomohisa Kanai[1], Tatsuya Shirahata[1, 2], Shunsuke Nakamori[1], Yota Koizumi[2], Eiichi Kodaira[1], Noriko Sato[1], Hiroyuki Fuchino[3], Noriaki Kawano[3], Nobuo Kawahara[3,4], Takayuki Hoshino[2], Kayo Yoshimatsu[3], Kobayashi Yoshinori[1, 2]*

[1]*School of Pharmacy, Kitasato University; 5–9–1 Shirokane, Minato-ku, Tokyo 108–8641, Japan.*

[2]*Kitasato University, Kitasato Institute Hospital, Oriental Medicine Therapy Center; 5–9–1 Shirokane, Minato-ku, Tokyo 108–8641, Japan.*

[3]*Research Center for Medicinal Plant Resources, National Institutes of Biomedical Innovation, Health and Nutrition; 1–2 Hachimandai, Tsukuba, Ibaraki 305– 0843, Japan*

[4]*The Kochi Prefectural Makino Botanical Garden;Godaisan, Kochi 781–8125, Japan.*

*Correspondence

Yoshinori Kobayashi, Ph.D., Japan; Tel.: 03-3444-6161 (2590); Fax: 03-5791-6171

E-mail: kobayashiy@pharm.kitasato-u.ac.jp

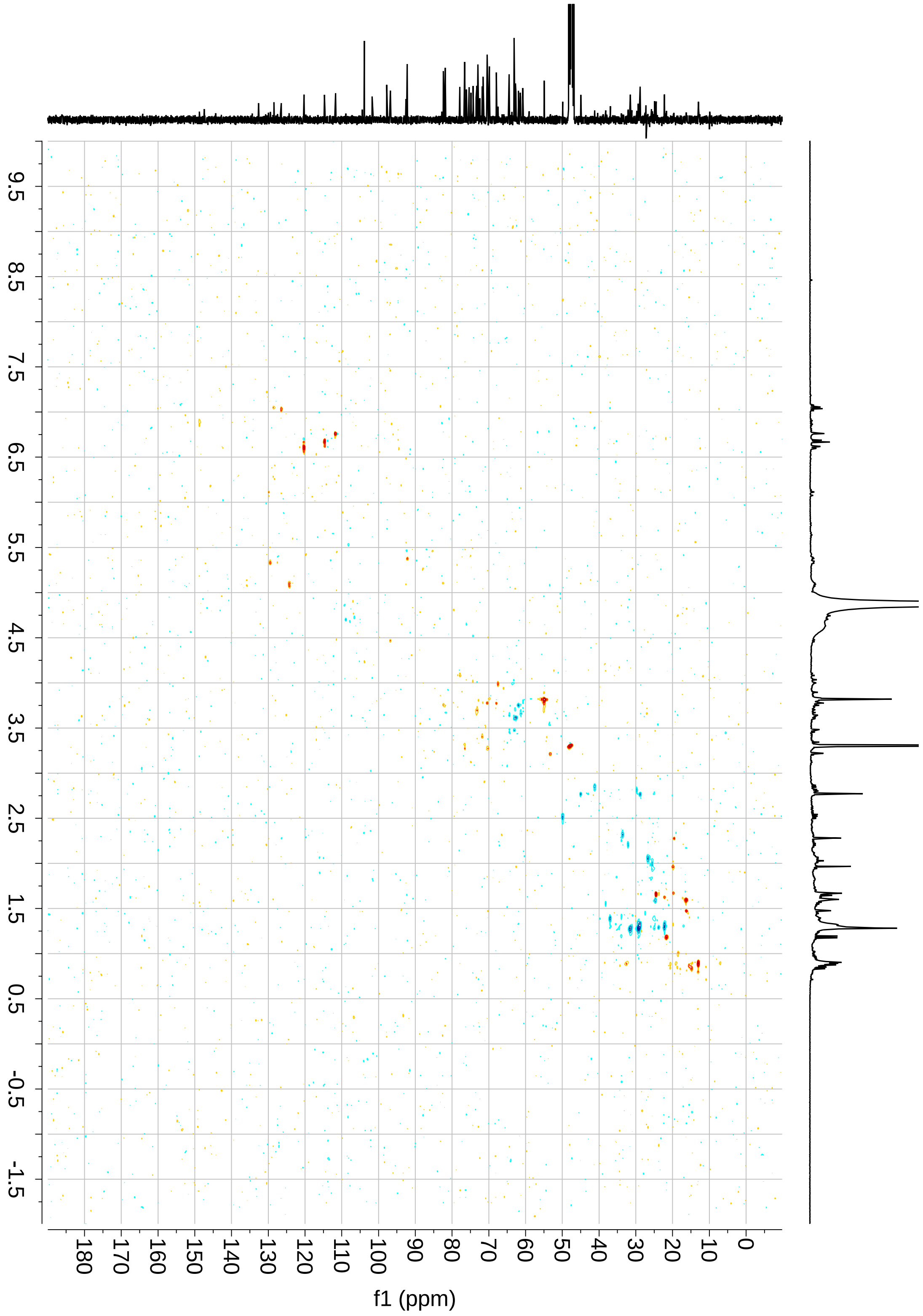

Fig. S1 HSQC spectrum of PGR (No. 6) CD_3OD extracted

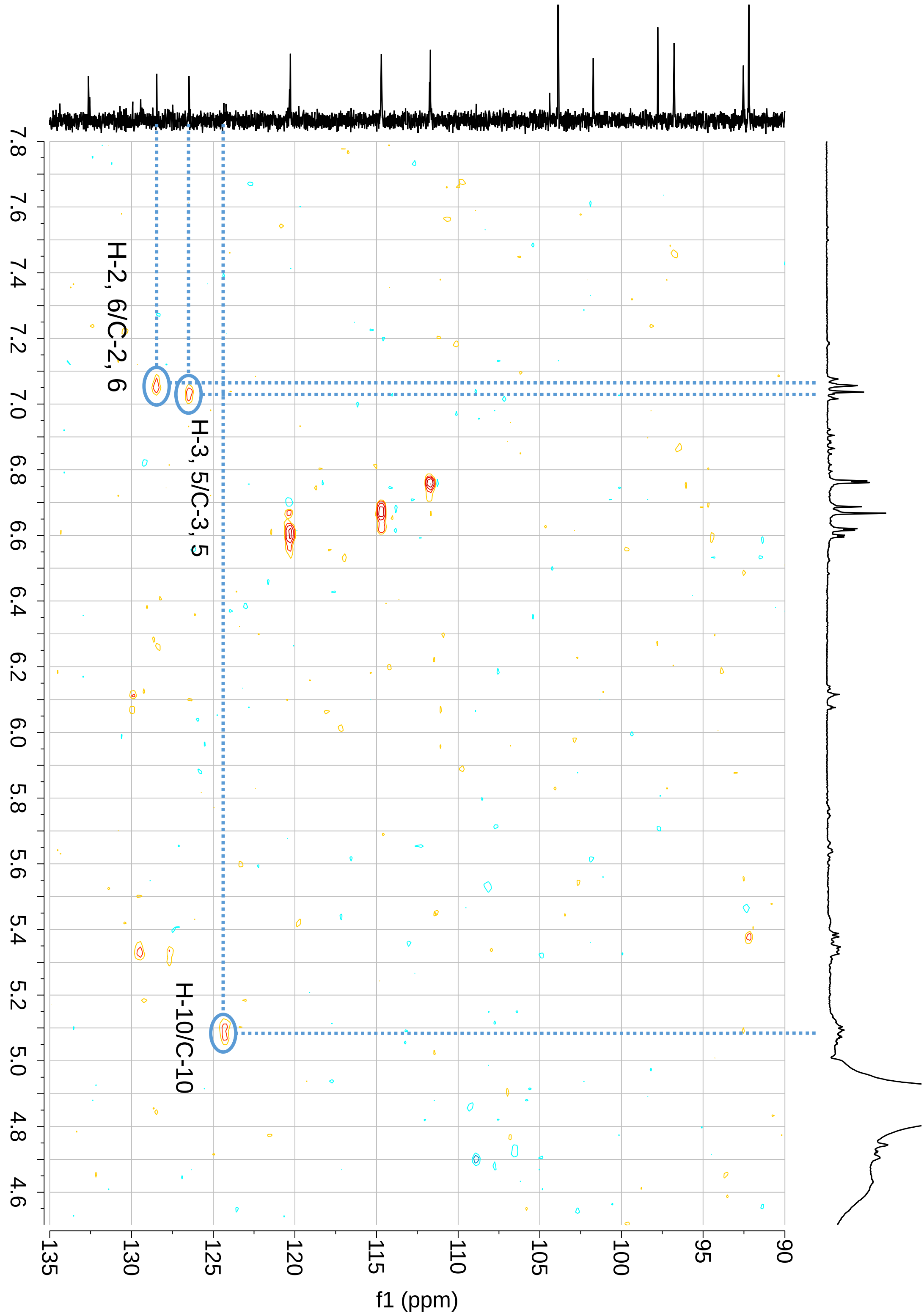

Fig. S2 Enlarged view of HSQC spectrum

(^{1}H-NMR; 7.80-4.50 ppm, ^{13}C-NMR; 135.0-90.0 ppm)

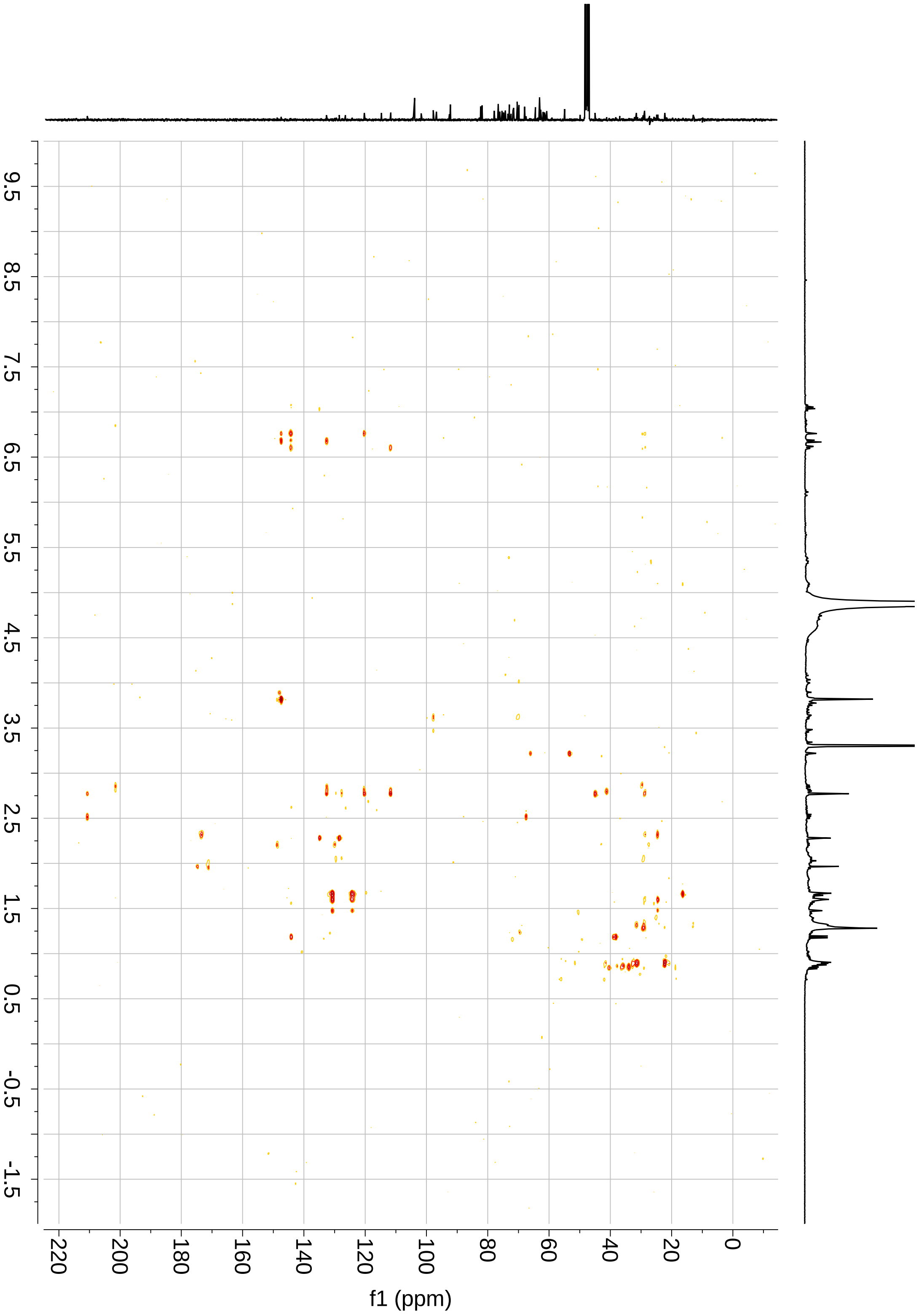

Fig. S3 HMBC spectrum of PGR (No. 6) CD_3OD extracted

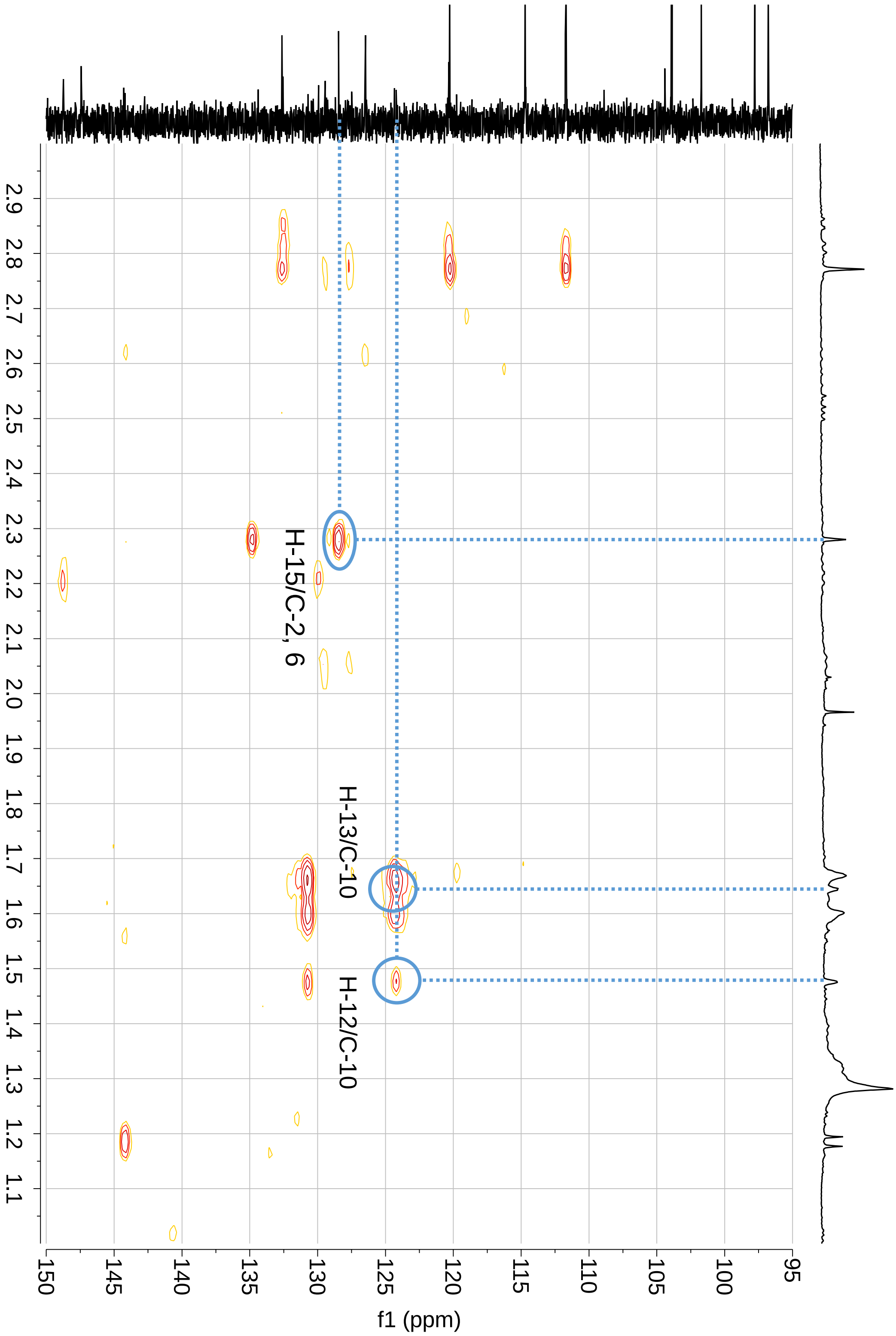

Fig. S4 Enlarged view of HMBC spectrum

(^{1}H-NMR; 3.00-1.00 ppm, ^{13}C-NMR; 150.0-95.0 ppm)

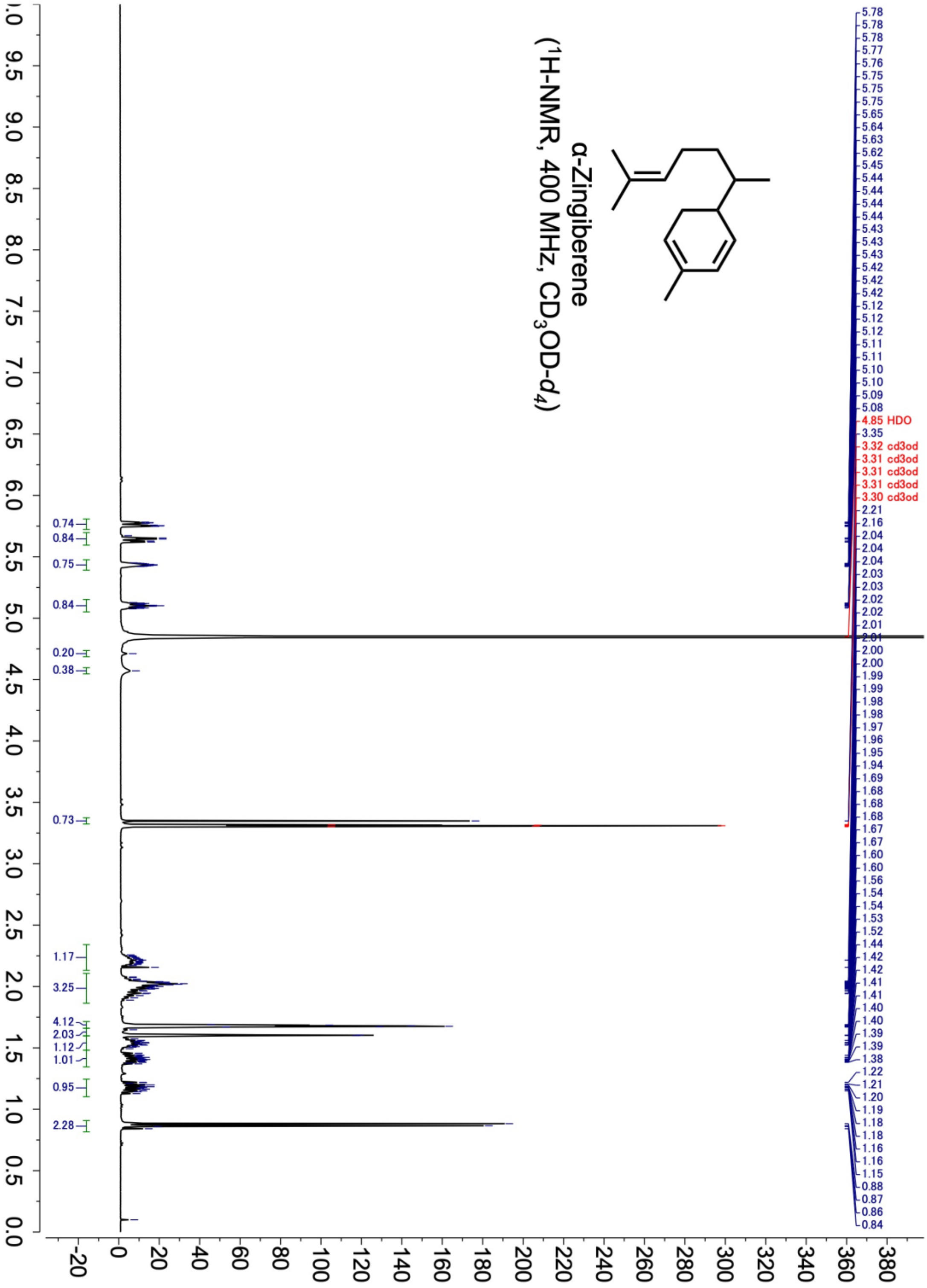

Fig. S5 ^{1}H-NMR chart of α-zingiberene

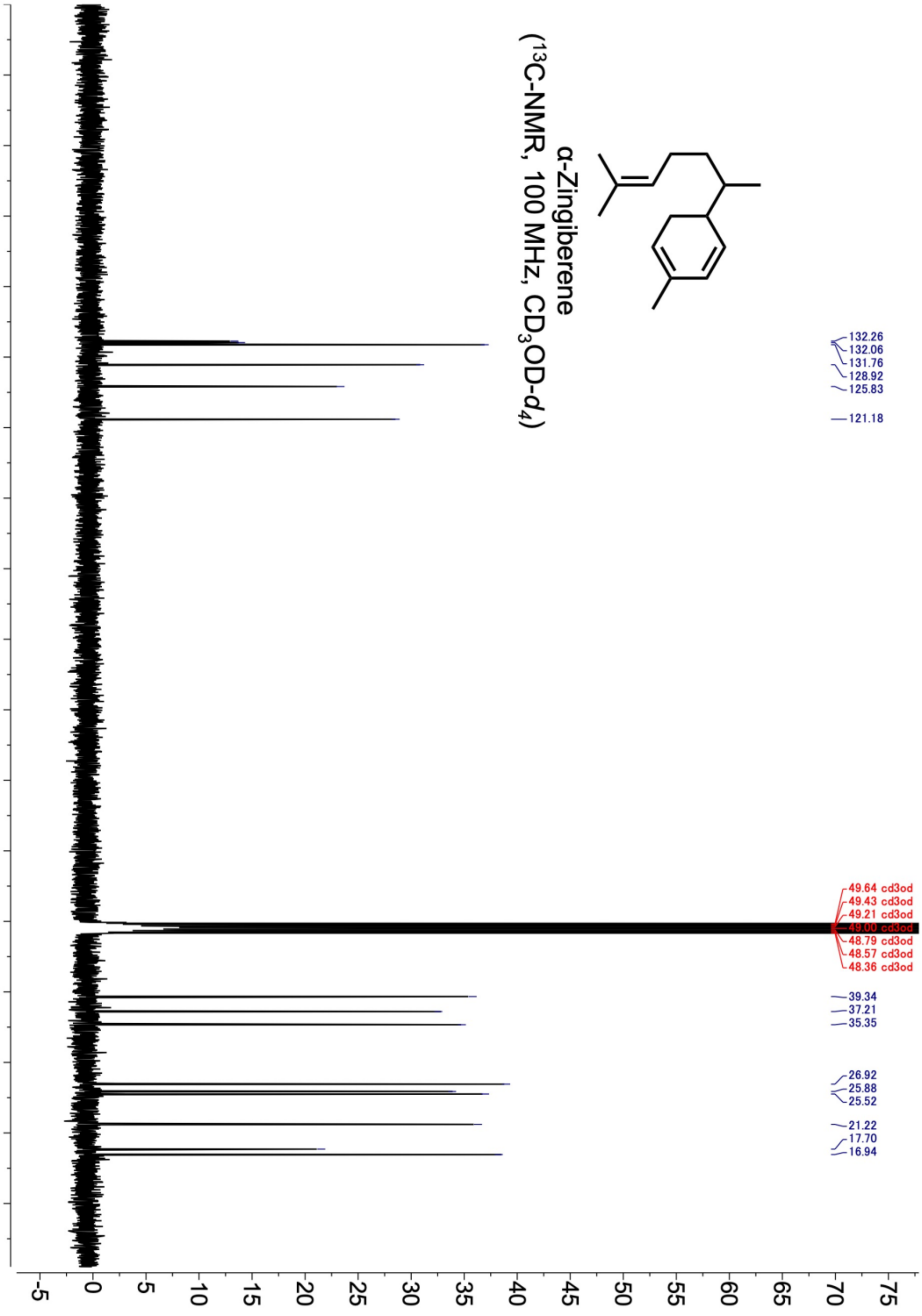

Fig. S6 ^{13}C-NMR Chart of α-zingiberene

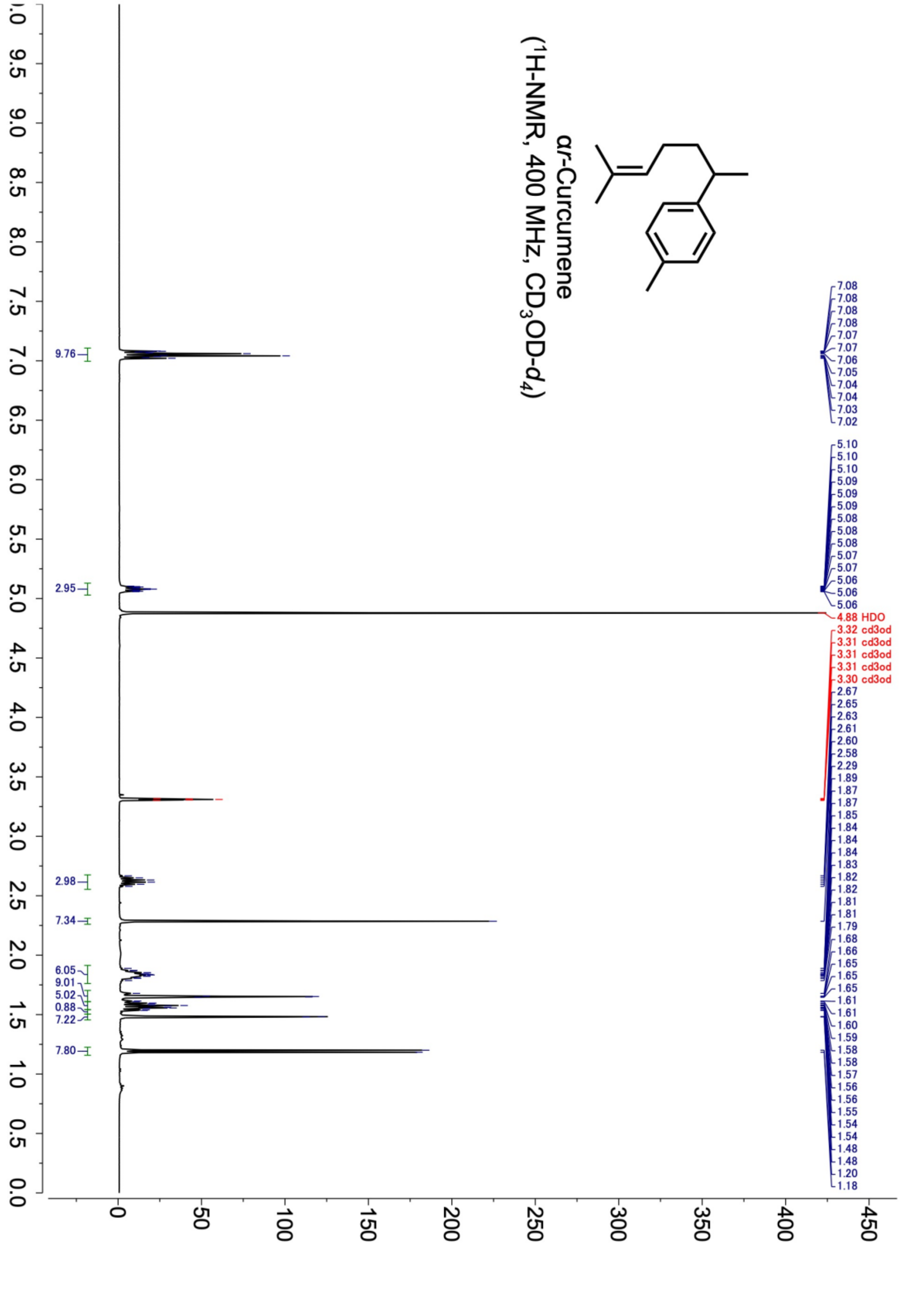

Fig. S3 ^{1}H-NMR Chart of *αr*-curcumene

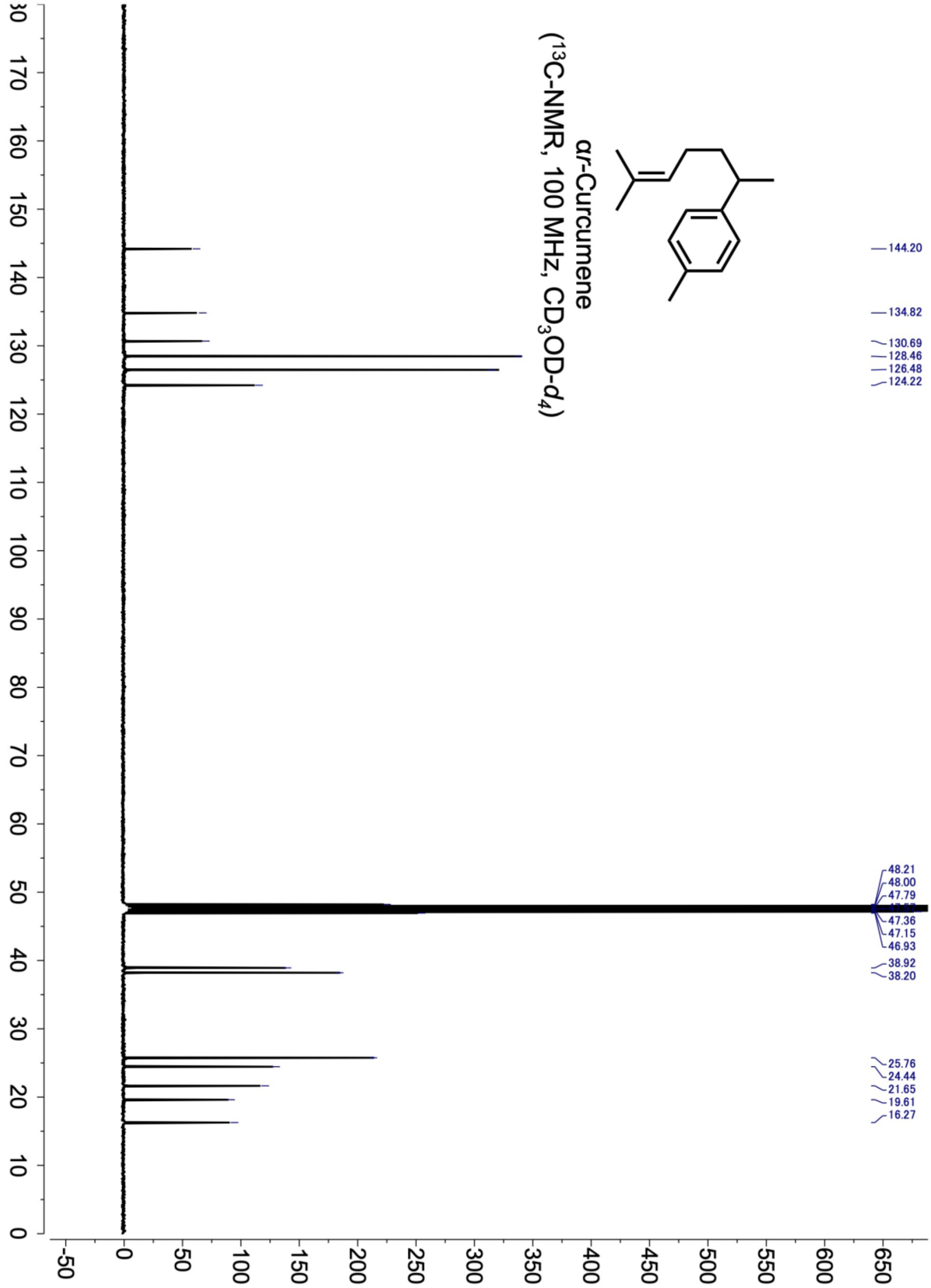

Fig. S8 ^{13}C-NMR Chart of *αr*-curcumene
